# Discontinuation of Anticoagulants and Occurrence of Bleeding and Thromboembolic Events in Vitamin K Antagonist Users with a Life-Limiting Disease

**DOI:** 10.1055/a-2524-5334

**Published:** 2025-04-04

**Authors:** Eva K. Kempers, Chantal Visser, Eric C. T. Geijteman, Jamilla Goedegebuur, Johanneke E. A. Portielje, Mette Søgaard, Anne Gulbech Ording, Carline van den Dries, Denise Abbel, Geert-Jan Geersing, Sarah J. Aldridge, Kate J. Lifford, Ashley Akbari, Sjef J. C. M. van de Leur, Melchior C. Nierman, Isabelle Mahé, Simon P. Mooijaart, Sebastian Szmit, Michelle Edwards, Simon I. R. Noble, Frederikus A. Klok, Qingui Chen, Suzanne C. Cannegieter, Marieke J. H. A. Kruip, SERENITY consortium

**Affiliations:** 1 Department of Hematology, Erasmus MC, Erasmus University Medical Center Rotterdam, Rotterdam, The Netherlands; 2 Department of Medical Oncology, Erasmus MC Cancer Institute, Rotterdam, The Netherlands; 3 Department of Medicine - Thrombosis and Haemostasis, Leiden University Medical Center, Leiden, The Netherlands; 4 Department of Clinical Epidemiology, Leiden University Medical Center, Leiden, The Netherlands; 5 Department of Medical Oncology, Leiden University Medical Center, Leiden, The Netherlands; 6 Department of Clinical Medicine, Danish Center for Health Services Research, Aalborg University, Aalborg University Hospital, Aalborg, Denmark; 7 Center for General Practice, Aalborg University, Aalborg, Denmark; 8 Department of General Practice and Nursing Science, Julius Center for Health Sciences and Primary Care, University Medical Center Utrecht, Utrecht University, Utrecht, The Netherlands; 9 Department of Gerontology and Geriatrics, Leiden University Medical Center, Leiden, The Netherlands; 10 LUMC Center for Medicine for Older People, LUMC, Leiden, The Netherlands; 11 Population Data Science, Faculty of Medicine, Health and Life Science, Swansea University, Swansea, United Kingdom; 12 Division of Population Medicine, Wales Centre for Primary and Emergency Care Research (PRIME Centre Wales), Cardiff University, Cardiff, United Kingdom; 13 Thrombosis Service, Isala Hospital Zwolle, Zwolle, The Netherlands; 14 Department of Thrombosis and Anticoagulation, Atalmedial Medical Diagnostic Centers, Amsterdam, The Netherlands; 15 Department of Internal Medicine, Paris Cité University, Assistance Publique des Hôpitaux de Paris, Louis Mourier Hospital, INSERM UMR_S1140, Innovations Thérapeutiques en Hémostase, F-CRIN INNOVTE Network, Colombes, France; 16 Department of Cardio-Oncology, Centre of Postgraduate Medical Education, Warsaw, Poland; 17 Division of Population Medicine, Marie Curie Palliative Care Research Centre, Cardiff University, Cardiff, Wales, United Kingdom

**Keywords:** anticoagulants, deprescriptions, thromboembolism, hemorrhage, advance care planning

## Abstract

**Background:**

Data on risks and benefits of long-term anticoagulants in patients with a life-limiting disease are limited. This cohort study aims to describe (dis)continuation of anticoagulants and incidences of bleeding and thromboembolic events in vitamin K antagonist (VKA) users with a life-limiting disease.

**Methods:**

Data from five Dutch anticoagulation clinics were linked to data from Statistics Netherlands and the Netherlands Cancer registry. Prevalent VKA users diagnosed with a pre-specified life-limiting disease between January 1, 2013 and December 31, 2019 were included and followed until December 31, 2019. Bleeding and thromboembolic events were identified by hospitalization data. Cumulative incidences of anticoagulant discontinuation, accounting for death as competing risk, and event rates for both anticoagulant exposed and unexposed person-years (PYs) were determined.

**Results:**

Among 18,145 VKA users (median age 81 years [IQR: 74–86], 49% females, median survival time 2.03 years [95%CI: 1.97–2.10]), the most common life-limiting diseases were heart disease (60.0%), hip fracture (18.1%), and cancer (13.5%). One year after diagnosis, the cumulative incidence of anticoagulant discontinuation was 14.0% (95%CI: 13.5–14.6). Over 80% of patients continued anticoagulant therapy until the last month before death, with median 14 days between discontinuation and death. Event rates per 100 PYs (95%CI) were comparable during anticoagulant use and after discontinuation for bleeding 2.6 (2.4–2.8) versus 2.1 (1.5–2.8), venous thromboembolism 0.2 (0.1–0.2) versus 0.4 (0.2–0.7), and arterial thromboembolism 3.1 (2.9–3.3) versus 3.3 (2.6–4.2).

**Conclusion:**

Most VKA users with a life-limiting disease continued anticoagulant treatment during their last phase of life, with similar rates of bleeding and thromboembolic events during use and after discontinuation.

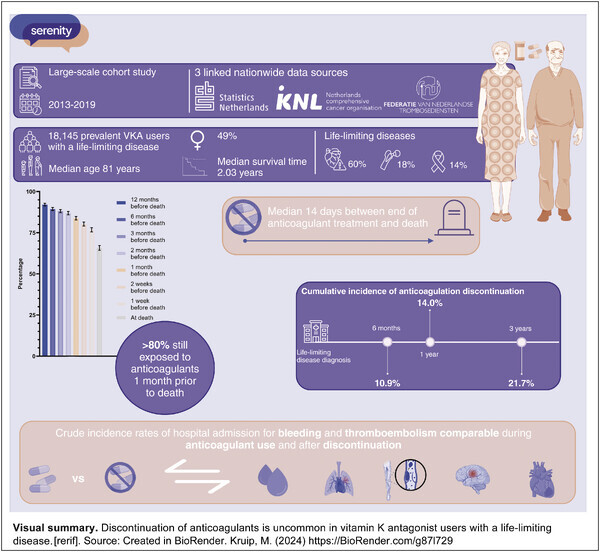

## Introduction

Anticoagulants are frequently prescribed for various indications. Direct oral anticoagulants (DOACs) are now the first choice for most common indications, such as venous thromboembolism (VTE), and prevention of thromboembolic complications in patients with non-valvular atrial fibrillation (AF). 1 2 3 However, in certain patient groups, including patients with a mechanical heart valve, 4 5 6 frail older patients with AF, 7 or patients with antiphospholipid syndrome, 3 vitamin K antagonists (VKAs) are still preferred over DOACs because of superior efficacy in preventing thromboembolic events and/or a safer bleeding risk profile. 3

In patients with a life-limiting disease, the drawbacks of continuing long-term anticoagulants, including frequent international normalized ratio (INR) monitoring during VKA therapy or heparin injections 8 and an increased risk of clinically relevant bleeding, 9 may outweigh its benefits. For these patients, advance care planning (ACP), which involves discussions about future care preferences between patients and their healthcare providers 10 and deprescribing drugs when potential harms outweigh benefits, 11 is now standard care. Yet, uncertainty among prescribing physicians about the outcomes of continuing or discontinuing drugs remains a barrier to deprescribing. 12 In the context of anticoagulant treatment, there is a lack of data and consensus on whether or not to continue anticoagulants in patients toward their end of life. 8 13 14 15 Patients with advanced life-limiting diseases, such as cancer, chronic kidney disease, or chronic obstructive pulmonary disease, face an increased risk of both bleeding and thromboembolic events, and this thromboembolic risk may increase if anticoagulant treatment is discontinued. 16 17 18 19 20 21 22 Furthermore, physicians might be reluctant to deprescribe anticoagulants because of the perceived thromboembolic risk associated with discontinuation. 13

Most previous studies on anticoagulant use during the last phases of life identified patients at the time of death and retrospectively analyzed their medical history backward in time. 14 23 24 This approach can introduce selection bias if anticoagulant use influences survival and precludes the use of time-to-event analyses. Consequently, the use, risks, and benefits of anticoagulants remain understudied in patients with life-limiting diseases. 8 Therefore, this large cohort study aims to (1) describe the use and discontinuation of VKAs and anticoagulant treatment overall, and (2) determine incidence rates of hospitalization for bleeding and thromboembolic events in VKA users with a life-limiting disease. This study is part of the “Towards Cancer Patient Empowerment for Optimal Use of Antithrombotic Therapy at the End of Life” (SERENITY) project. 25

## Methods

### Setting and Data Sources

Our cohort study used data from five large Dutch anticoagulation clinics, linked on an individual-level to data from Statistics Netherlands and the Netherlands Cancer Registry (NCR). Anticoagulation clinics monitor the VKA therapy of patients living in well-defined geographical areas in the Netherlands and provide detailed data on VKA treatment, including start and end dates of VKA therapy, treatment indications, and INR measurements. 26 Data from these clinics were linked to data from Statistics Netherlands by sex, date of birth, postal code, and last known date to be alive.

Statistics Netherlands provides nationwide data on personal characteristics, 27 diagnoses made during hospital admissions in Dutch hospitals, 28 29 30 31 cause of death, 32 and date of death. 33 Diagnoses in the Dutch Hospital Data (DHD) registry are coded according to the International Classification of Diseases (ICD) (ICD-9 for diagnoses made from 2010 to 2012, and ICD-10 thereafter). Additionally, data on outpatient medication prescriptions covered by the Dutch statutory basic medical insurance were also obtained from Statistics Netherlands. 34 These data included the year of prescription and Anatomical Therapeutic Chemical (ATC) code. For anticoagulants specifically, more detailed prescription data were available, including dispending date and type of anticoagulant (i.e., VKA, DOAC, or heparin group). However, data on medications received in hospitals and nursing homes were not available.

Data from the NCR were linked to Statistics Netherlands by sex, date of birth, and postal code. The NCR data are provided by the Netherlands Comprehensive Cancer Organization and comprises individual-level data on newly diagnosed cancer patients in the Netherlands. 35 This registry includes information on cancer diagnosis, tumor stage according to the TNM classification, 36 and whether a patient had received surgery, systemic chemotherapy, and/or radiotherapy immediately after their index diagnosis. All data were anonymized, and each individual was assigned a unique identification code by the Statistics Netherlands. A detailed description of the data sources is provided in Supplementary Methods (available in the online version).

### Study Population

The source population comprised VKA users who were treated at one of the participating anticoagulation clinics between 2013 and 2019. From this population, we included VKA users who were hospitalized with a pre-specified life-limiting disease or who received a severe cancer diagnosis. Life-limiting diseases were defined according to the definition of a severe medical condition by Kelley et al: “ *a diagnosis that carries an increased risk of mortality, hospitalization and emergency room visits* .” 37 To identify a cohort of patients with limited life expectancy, we restricted to diseases with median survival times of 2 to 4 years, which were predefined based on nationwide Dutch data. The following non-cancer diseases, derived from the Statistics Netherlands data, were included: liver disease, dementia, heart disease, lung disease, diabetes mellitus with complications, and hip fracture (in patients >70 years of age) ( Supplementary Tables S1 and S2 , available in the online version). Data on cancer diagnoses and severity were derived from the NCR. Similar to non-cancer diseases, we restricted to cancer types with a median survival time of 3 years or less at the time of diagnosis. These included all stages of pancreatic, brain, and hepatobiliary cancer and unknown primary tumor; stage III and IV cervical, bladder, ovarian, lung, esophageal, and gastric cancer and neuro-endocrine tumors (NETs); and stage IV of endometrial, breast, and colorectal cancer ( Supplementary Tables S3 and S4 , available in the online version). The index date was defined as the date of first hospitalization with a life-limiting disease or the date of the first severe cancer diagnosis during the study period, whichever came first. VKA users were defined as patients with two registered INR measurements in the 6 months preceding the index date, with at least one in the 3 months preceding the index date. Patients who did not meet this definition on their index date were not classified as a VKA user and therefore excluded. Patients were followed until the end of the study period (December 31, 2019) or their date of death, whichever occurred first.

### Baseline Characteristics

Information on age, sex, immigration background, and standardized household income was collected at index date. Discharge codes from the DHD registry were used to collect information on comorbidities diagnosed within the 3 years before the index date ( Supplementary Table S5 , available in the online version). We also collected data on outpatient prescriptions of steroids, antidepressants, antacids, antihypertensive, lipid-lowering, anti-inflammatory, and antiplatelet drugs, and the presence of polypharmacy in the calendar year of the index date ( Supplementary Table S6 , available in the online version). To account for changes within pharmacological subgroups, polypharmacy was defined as ≥5 registered prescriptions of different drug types at the therapeutic level (2nd) of the ATC classification 38 based on all dispensed outpatient drugs in the calendar year of the index date.

### Exposure to VKAs and Other Anticoagulants

Exposure to VKAs during follow-up was primarily derived from INR records and the start and end dates of VKA therapy, as registered by anticoagulation clinics. Continuous VKA exposure was defined as subsequent INR measurements ≤8 weeks apart, consistent with guidelines used by Dutch anticoagulation clinics. 39 To account for switching to non-VKA anticoagulants or transfers to non-participating anticoagulation clinics, we used data on dispensed prescriptions for VKAs, DOACs, and low-molecular-weight heparins (LMWHs) covering 2012 to 2020 from Statistics Netherlands. This allowed us to examine VKA and anticoagulant exposure after the registered VKA end dates from the anticoagulation clinics. Both VKA and anticoagulant exposure were modelled by constructing treatment periods of person–time exposed according to dispensed prescriptions, considering only prescriptions after the VKA end dates recorded by anticoagulation clinics. Construction of treatment periods for the different anticoagulant types is illustrated in Supplementary Figs. S1 and S2 (available in the online version) and described in the Supplementary Methods (available in the online version). Anticoagulant treatment periods could consist of VKA, DOAC, or LMWH therapy.

### Study Outcomes

For the first objective, the study outcomes were discontinuation of VKAs and discontinuation of anticoagulant treatment overall. To address this, the last VKA and anticoagulant treatment periods for each patient were identified. Patients were considered to have discontinued VKAs and/or anticoagulants if the end date of their last treatment period occurred before their end of follow-up (date of death or December 31, 2019, whichever occurred first). In addition, we calculated the proportion of days covered (PDC) with anticoagulants for each patient, defined as the ratio of days treated with anticoagulants following the index date and the total number of follow-up days.

For the second objective, the study outcomes were hospital admissions for major and non-major clinically relevant bleeding, VTE, and arterial thromboembolism (ATE), including stroke and transient ischemic attacks (TIA), myocardial infarction, and other ATE. These outcomes were identified from the DHD registry and restricted to primary or main diagnoses during hospitalization ( Supplementary Methods and Supplementary Table S7 , available in the online version). For each outcome, separately, patients were followed until first event of interest, death, or end of follow-up, whichever occurred first, regardless of anticoagulant treatment.

### Statistical Analyses

For descriptive analyses, continuous variables were presented as mean ± standard deviation (SD) or median and interquartile range (IQR). Categorical variables were presented as numbers with percentages. Survival curves and median survival time were estimated by the Kaplan-Meier estimator, and median follow-up time was calculated by the reverse Kaplan-Meier. 40 For the first objective, the cumulative incidences of VKA and anticoagulant discontinuation were calculated with death as a competing risk, based on the Fine and Gray method. 41 We also estimated crude incidence rates of VKA and anticoagulant discontinuation as events per 100 person-years (PYs) with corresponding 95% confidence intervals (95%CI). Additionally, for patients who died during follow-up, we described the use of anticoagulants during the last period of life. For this analysis, we categorized patients into subgroups according to their available follow-up time from index date to the date of death: at least 365 days, 180–364 days, and 90–179 days. Point estimates with corresponding 95%CI for the proportion of patients exposed to anticoagulants were calculated for different time points in the period before death, ranging from 1 week to 1 year before death.

Similarly, for the second objective, cumulative incidences of first bleeding and thromboembolic events, separately, were calculated with death as competing risk. In addition, incidence rates per 100 PYs of first bleeding and thromboembolic events were estimated stratified by anticoagulant exposure. Observation time was categorized as anticoagulant exposed and unexposed, and events were assigned to the relevant exposure stratum at the time of the event. All analyses were stratified by cancer versus non-cancer disease. Statistical analyses were performed in SPSS® Statistics (IBM Corp. Released 2017. IBM SPSS Statistics for Windows, Version 25.0. Armonk, NY: IBM Corp.) and R 4.4.0. 42 R packages used are listed in the Supplementary Methods .

### Sensitivity Analyses

To examine the impact of our assumptions about treatment duration, we performed several sensitivity analyses when constructing treatment periods for both VKAs and anticoagulants based on dispensed prescriptions ( Supplementary Methods ). To examine possible time-related bias due to misclassification of anticoagulant exposure and/or outcome, the period exposed to anticoagulants was extended by 7 days when calculating incidence rates of bleeding and thromboembolic events stratified by anticoagulant exposure.

## Results

### Baseline Characteristics

**Fig. 1:**
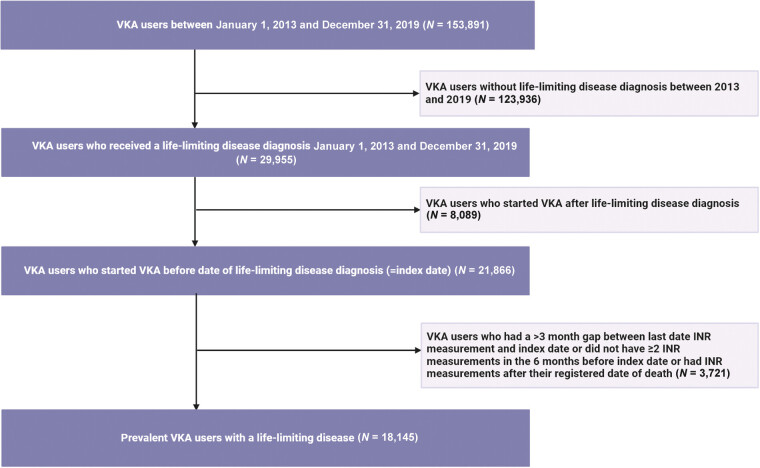
Flowchart of cohort selection. This figure presents the flowchart for the selection of the study cohort. A life-limiting disease was defined according to the definition of a severe medical condition by Kelley et al as “a diagnosis that carries an increased risk of mortality, hospitalization and emergency room visits.” 37 INR, international normalized ratio; VKA, vitamin K antagonist. [rerif]. Source: Created in BioRender. Kruip, M. (2024) https://BioRender.com/e31d202 .

Between 2013 and 2019, 18,145 VKA users had a hospital admission for a life-limiting disease or received a severe cancer diagnosis ( Fig. 1 ). At index date, the median age of the cohort was 81 years (IQR 74–86), 49% was female, and 86% was native Dutch ( Table 1 and Supplementary Table S8 , available in the online version). In addition, 45% had one or more comorbidities, and almost all patients (94.6%) fulfilled our definition of polypharmacy. The most common life-limiting diseases were heart disease (60.0%), hip fracture (18.1%), and cancer (13.5%). AF or other arrhythmias were the most common indication for VKA therapy (78.7%); other indications included cardiomyopathy (5.8%), VTE (5.6%), and mechanical heart valves (5.3%). Among patients with cancer, the most common cancer type was lung (39.1%), followed by pancreatic (11.6%) and colorectal (10.8%) cancer ( Table 1 and Supplementary Table S9 , available in the online version).

**Table 1:** Baseline characteristics

	Total ( *N* = 18,145)	Cancer ( *N* = 2,457)	Non-cancer ( *N* = 15,688)
**Demographics**			
Female sex, No. (%)	8,841 (48.7)	893 (36.3)	7,948 (50.7)
Age at index date, median (Q1, Q3), years	81.0 (74.0, 86.0)	77.0 (70.0, 82.0)	81.0 (74.0, 87.0)
Immigration background ^a^ , No. (%)			
Dutch background	15,649 (86.2)	2,199 (89.5)	13,450 (85.7)
First-generation migration background	1,781 (9.8)	159 (6.5)	1,622 (10.3)
Second-generation migration background	715 (3.9)	99 (4.0)	616 (3.9)
**Year of diagnosis, No. (%)**			
2013	2,264 (12.5)	276 (11.2)	1,988 (12.7)
2014	2,596 (14.3)	362 (14.7)	2,234 (14.2)
2015	2,739 (15.1)	366 (14.9)	2,373 (15.1)
2016	2,738 (15.1)	362 (14.7)	2,376 (15.1)
2017	2,977 (16.4)	408 (16.6)	2,569 (16.4)
2018	2,536 (14.0)	345 (14.0)	2,191 (14.0)
2019	2,295 (12.6)	338 (13.8)	1,957 (12.5)
** Type of life-limiting disease b , No. (%) **			
Cancer	2,457 (13.5)	2,457 (100)	0 (0)
ILD	107 (0.6)	0 (0)	107 (0.7)
Liver disease	164 (0.9)	0 (0)	164 (1.0)
COPD	547 (3.0)	0 (0)	547 (3.5)
Dementia	387 (2.1)	0 (0)	387 (2.5)
Diabetes mellitus (complicated)	327 (1.8)	0 (0)	327 (2.1)
Hip fracture	3,277 (18.1)	0 (0)	3,277 (20.9)
Heart disease	10,879 (60.0)	0 (0)	10,879 (69.3)
**Type of cancer, No. (%)**			
Esophagus	165 (0.9)	165 (6.7)	NA
Stomach	104 (0.6)	104 (4.2)	NA
Colorectal	265 (1.5)	265 (10.8)	NA
Hepatobiliary	170 (0.9)	170 (6.9)	NA
Pancreas	284 (1.6)	284 (11.6)	NA
Bronchus and lung	960 (5.3)	960 (39.1)	NA
Breast	49 (0.3)	49 (2.0)	NA
Ovary	53 (0.3)	53 (2.2)	NA
Other female genital organs	28 (0.2)	28 (1.1)	NA
Bladder	115 (0.6)	115 (4.7)	NA
Brain	66 (0.4)	66 (2.7)	NA
Other c	16 (0.1)	16 (0.7)	NA
Unknown primary or multiple tumors	182 (1.0)	182 (7.4)	NA
** TNM stage d , No. (%) **			
I	70 (0.4)	70 (2.8)	NA
II	64 (0.4)	64 (2.6)	NA
III	572 (3.1)	572 (23.3)	NA
IV	1,382 (7.6)	1,382 (56.2)	NA
* Unknown*	31 (0.2)	31 (1.3)	NA
* Not applicable*	338 (1.9)	338 (13.8)	NA
** Registered indications for VKA therapy e , No. (%) **			
Mechanical heart valve	960 (5.3)	101 (4.1)	859 (5.5)
Biological valve and other heart surgery	381 (2.1)	33 (1.3)	348 (2.2)
Atrial fibrillation and other arrhythmias	14,282 (78.7)	1,757 (71.5)	12,525 (79.8)
Decompensation cordis and valvular disease	371 (2.0)	22 (0.9)	349 (2.2)
Cardiomyopathy	1,054 (5.8)	81 (3.3)	973 (6.2)
Cerebral vascular disease	296 (1.6)	31 (1.3)	265 (1.7)
Arterial embolism	181 (1.0)	32 (1.3)	149 (0.9)
Peripheral arterial disease	156 (0.9)	24 (1.0)	132 (0.8)
Coronary syndrome and interventions	439 (2.4)	60 (2.4)	379 (2.4)
Vascular surgery	400 (2.2)	93 (3.8)	307 (2.0)
VTE	1,024 (5.6)	304 (12.4)	720 (4.6)
Other	187 (1.0)	30 (1.2)	157 (1.0)
**INR target range, No. (%)**			
2.0–3.0	2,606 (14.4)	325 (13.2)	2,281 (14.5)
2.5–3.5	11,929 (65.7)	1,578 (64.2)	10,351 (66.0)
3.0–4.0	2,136 (11.8)	250 (10.2)	1,886 (12.0)
Other	117 (0.6)	17 (0.7)	100 (0.6)
* Unknown*	1,357 (7.5)	287 (11.7)	1,070 (6.8)
**Type of VKA, No. (%)**			
Acenocoumarol	13,871 (76.4)	1,769 (72.0)	12,102 (77.1)
Phenprocoumon	4,269 (23.5)	688 (28.0)	3,581 (22.8)
** ≥1 comorbidity present at index date f , No. (%) **	8,195 (45.2)	956 (38.9)	7,239 (46.1)
** Medication use at index date g , No. (%) **			
Antiplatelet drugs	2,853 (15.7)	261 (10.6)	2,592 (16.5)
Antihypertensives	16,964 (93.5)	2,178 (88.6)	14,786 (94.3)
Anti-inflammatory (non-steroidal)	1,748 (9.6)	388 (15.8)	1,360 (8.7)
Anti-depressants	2,405 (13.3)	312 (12.7)	2,093 (13.3)
Lipid-lowering	9,828 (54.2)	1,345 (54.7)	8,483 (54.1)
Steroids	5,244 (28.9)	1,077 (43.8)	4,167 (26.6)
Antacids	11,572 (63.8)	1,531 (62.3)	10,041 (64.0)
Polypharmacy h	17,168 (94.6)	2,353 (95.8)	14,815 (94.4)
Polypharmacy i (excluding antithrombotic agents)	16,802 (92.6)	2,305 (93.8)	14,497 (92.4)
* Unknown*	467 (2.6)	27 (1.1)	440 (2.8)

Abbreviations: COPD, chronic obstructive pulmonary disease; ILD, interstitial lung disease; INR, international normalized ratio; IQR, interquartile range; TNM, tumor, nodes and metastases; VKA, vitamin K antagonist; VTE, venous thromboembolic event.

a Immigration data were collected from Statistics Netherlands. Dutch background is defined as having both parents born in the Netherlands. First-generation migration background is defined as being born abroad with at least one parent who was born abroad; second-generation migration background is defined as being born in the Netherlands with at least one parent who was born abroad.

b A life-limiting disease was defined according to the definition of a severe medical condition by Kelley et al as “a diagnosis that carries an increased risk of mortality, hospitalization and emergency room visits.” 37 These diseases were identified by ICD-10 codes of diagnoses registered as either main or primary diagnosis of the hospital admission or registered cancer diagnosis by the Netherlands Cancer Registry. Patients were classified according to the first type of life-limiting disease that occurred during the study period.

c Other cancer types included (neuro-endocrine) tumors located in the small intestine, peritoneum, or retroperitoneum.

d TNM stage was based on the 7th edition 57 of the tumor-node-metastasis cancer staging system for index dates between 2013 and 2016 and the 8th edition 36 of the tumor-node-metastasis cancer staging system for index dates between 2017 and 2019.

e All treatment indications for vitamin K antagonist treatment that have been registered until the date of data export and were identified from the Dutch anticoagulation clinics. One or more indications can be present.

f Comorbidities were identified by examining data on hospitalizations within 3 years before the index date using ICD-10 codes and ICD-9 codes, restricting to main or primary diagnosis of hospital admission. One or more comorbidities can be present. The following comorbidities were identified: autoimmune disease or immune deficiency, thyroid disease, COPD, asthma or other chronic lung disease, major and clinically relevant bleeding, venous thromboembolism, arterial thromboembolism, stroke, myocardial infarction, anemia, coagulopathy, heart failure, valvular heart disease, atrial fibrillation, atherosclerosis, peripheral arterial disease, diabetes mellitus, hypertension, and kidney and liver disease.

g Medication use at index date was identified by examining outpatient medication prescriptions covered by the Basic Dutch Health Insurance based on ATC codes in the calendar year of the index date.

h Polypharmacy was defined as ≥5 different drug types in the calendar year of the index date, at the therapeutic (2nd) level of the ATC classification.

i Polypharmacy was defined as ≥5 different drug types in the calendar year of the index date, at the therapeutic (2nd) level of the ATC classification, excluding the therapeutic class antithrombotic agents (B01).

### Follow-up and All-cause Mortality

The overall median follow-up time (IQR) was 3.59 years (1.95–5.22), with 3.41 years (2.06–5.07) in patients with cancer and 3.60 years (1.95–5.22) in patients with non-cancer diseases ( Supplementary Table S10 , available in the online version). During follow-up, 10,948 patients died, with an overall median survival time (95%CI) of 2.03 years (1.97–2.10) ( Supplementary Fig. S3A , available in the online version). Patients with cancer had shorter survival time than patients with non-cancer diseases (0.35 years [95%CI 0.32–0.38] and 2.50 years [95%CI 2.43–2.59], respectively) ( Supplementary Fig. S3B , available in the online version).

According to INR records from the anticoagulation clinics, 5,893 (32.5%) patients had at least one registered VKA end date during follow-up, and 382 (2.1%) patients interrupted their VKA treatment with a median duration of treatment interruption of 231 days (IQR 161–475). Despite having a VKA end date registered by the anticoagulation clinics, 10.9% of all VKA treatment periods ( *N* = 18,560) indicated that patients continued to be exposed to anticoagulants after their VKA end date according to dispensing data from Statistics Netherlands. Specifically, 0.7% of patients ( *N* = 129) interrupted their VKA therapy according to INR records, whereas these patients continued to be exposed to anticoagulants according to dispensed prescriptions.

### VKA and Anticoagulation Discontinuation

**Fig. 2:**
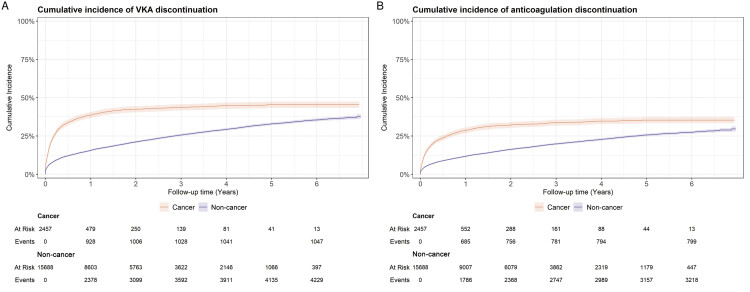
Cumulative incidences of vitamin K antagonist (VKA) and anticoagulant treatment discontinuation stratified by cancer versus non-cancer diseases. This figure illustrates the cumulative incidences of VKA ( **A** ) and anticoagulant ( **B** ) treatment discontinuation, stratified by whether patients had a cancer or non-cancer life-limiting disease, and accounting for the competing risk of death. The number of events at 5 years is masked according to privacy policy of Statistics Netherlands. AC, anticoagulation. VKA, vitamin K antagonist.

At 3 months after index date, the cumulative incidence (95%CI) of VKA discontinuation was 12.0% (11.5–12.5), which increased to 28.1% (27.4–28.8) at 3 years of follow-up ( Table 2 ). Some patients switched to other types of anticoagulants, as illustrated by the lower cumulative incidence of overall anticoagulant treatment discontinuation, which ranged from 8.6% (8.2–9.0) at 3 months to 21.7% (21.1–22.4) at 3 years. The VKA and anticoagulant discontinuation rates per 100 PYs (95%CI) were 17.9 (17.4–18.4) and 12.9 (12.5–13.3), respectively. Throughout follow-up, the incidence of discontinuing both VKAs and overall anticoagulant treatment was higher in patients with cancer compared with non-cancer diseases ( Fig. 2 ), with an anticoagulant discontinuation rate per 100 PYs (95%CI) of 42.6 (39.7–45.7) in patients with cancer versus 11.0 (10.6–11.4) in patients with non-cancer diseases ( Table 2 and Supplementary Table S11 , available in the online version). The overall mean PDC (SD) with anticoagulants was 91.8% (22.2), with 88.0% of patients classified as adherent to anticoagulants defined by a PDC >80% ( Supplementary Table S12 , available in the online version).

**Table 2:** Cumulative incidence and incidence rate of VKA and anticoagulant treatment discontinuation

	VKA discontinuation				Anticoagulation discontinuation			
	IR/100 PY (95%CI)	6-month cumulative incidence % (95%CI)	1-year cumulative incidence % (95%CI)	3-year cumulative incidence % (95%CI)	IR/100 PY (95%CI)	6-month cumulative incidence % (95%CI)	1-year cumulative incidence % (95%CI)	3-year cumulative incidence % (95%CI)
**Total**	17.9 (17.4–18.4)	15.0 (14.5–15.5)	18.7 (18.1–19.3)	28.1 (27.4–28.8)	12.9 (12.5–13.3)	10.9 (10.4–11.3)	14.0 (13.5–14.6)	21.7 (21.1–22.4)
**Type of life-limiting disease**								
Cancer	63.1 (59.3–67.0)	33.7 (31.9–35.6)	38.7 (36.7–40.6)	43.7 (41.7–45.8)	42.6 (39.7- 45.7)	23.8 (22.1–25.5)	28.7 (26.9–30.5)	33.7 (31.7–35.6)
Non-cancer	15.2 (14.8–15.7)	12.1 (11.6–12.6)	15.6 (15.0–16.2)	25.6 (24.9–26.4)	11.0 (10.6–11.4)	8.9 (8.4–9.3)	11.8 (11.2–12.3)	19.8 (19.2–20.5)
**Sex**								
Females	18.0 (17.3–18.7)	14.9 (14.2–15.7)	19.0 (18.2–19.8)	29.0 (28.0–30.0)	13.3 (12.7–13.9)	11.1 (10.5–11.8)	14.5 (13.7–15.2)	22.9 (21.9–23.8)
Males	17.8 (17.2–18.5)	15.1 (14.3–15.8)	18.5 (17.7–19.3)	27.2 (26.3–28.2)	12.5 (12.0–13.1)	10.7 (10.0–11.3)	13.6 (12.9–14.4)	20.6 (19.7–21.5)

Abbreviations: CI, confidence interval; IR, incidence rate; PY, person-years; VKA, vitamin K antagonist.

Note: Cumulative incidences were computed taking the competing risk of death into account. Crude incidence rates (IR) were estimated as events per 100 person-years (PY).

### Anticoagulant Use during the Last Phase of Life

**Fig. 3:**
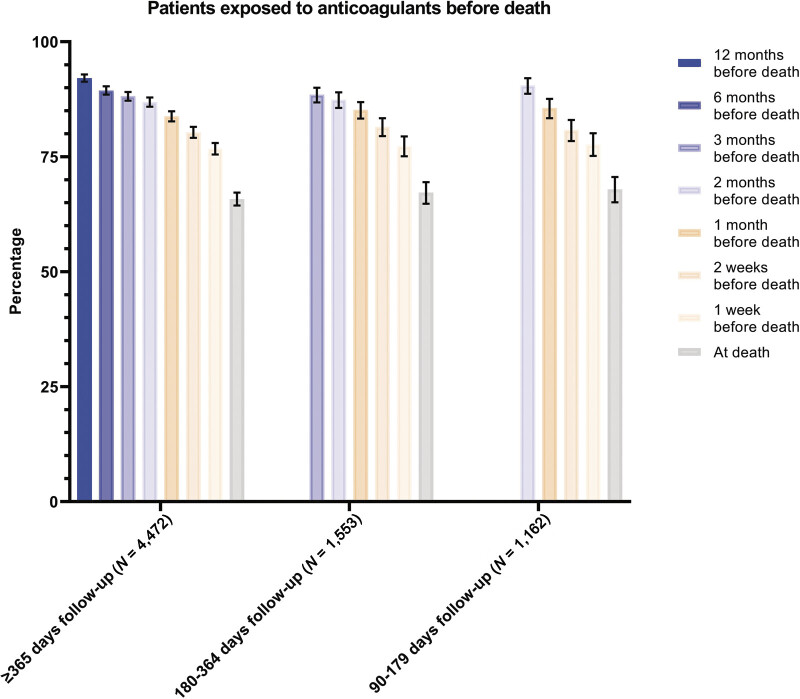
Percentage of patients exposed to anticoagulants before death. This figure displays the percentage of patients exposed to anticoagulants, along with corresponding 95% confidence intervals, at different time points before death. The analysis was stratified according to the amount of follow-up time between index date and date of death, and was restricted to patients who died during follow-up.

On their date of death, 69.2% of patients were still exposed to anticoagulants according to the constructed anticoagulant treatment periods. Among patients with a follow-up time of at least 365 days, the proportion exposed to anticoagulants (95%CI) decreased from 92.1% (91.2–92.8) at 1 year before death to 83.8% (82.7–84.9) at 1 month, and 76.8% (75.5–78.0) at 1 week before death ( Fig. 3 and Supplementary Fig. S4 , available in the online version). The median time gap between the constructed end of anticoagulant treatment and death among patients who discontinued anticoagulants before death ( *N* = 3,372) was 14 days (IQR 4–63) ( Supplementary Fig. S5 , available in the online version).

### Incidence of Bleeding and Thromboembolic Events

**Fig. 4:**
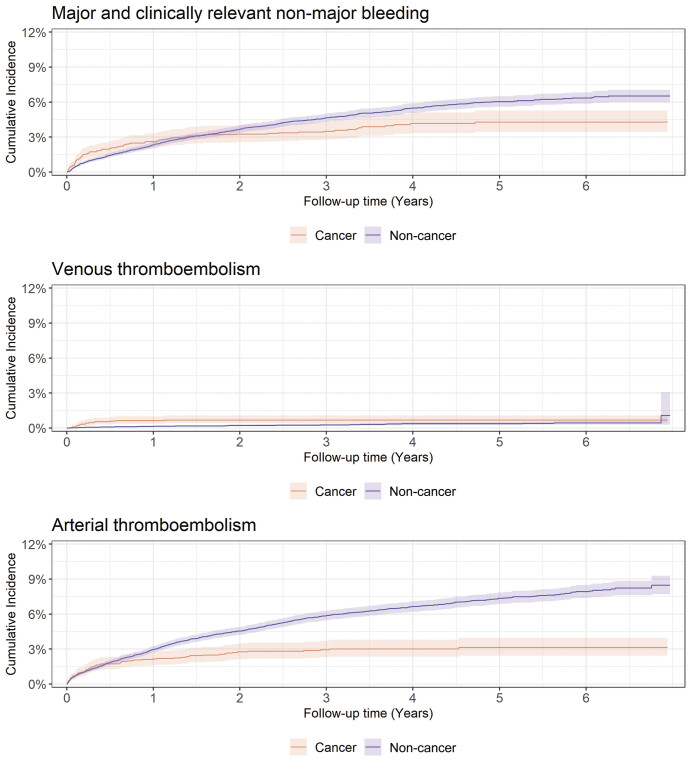
Cumulative incidences of bleeding and thromboembolic events stratified by cancer versus non-cancer diseases. This figure shows the cumulative incidences of first bleeding and thromboembolic events accounting for the competing risk of death. Venous thromboembolism included pulmonary embolism, deep vein thrombosis, cerebral sinus thrombosis, portal vein thrombosis, and other types of venous thromboembolism. Arterial thromboembolism included ischemic stroke, transient ischemic attack, myocardial infarction, and other types of arterial thromboembolism.

Point estimates of the incidence rate per 100 PYs (95%CI) of hospital admission for major or clinically relevant bleeding were slightly higher during anticoagulant use than after discontinuation: 2.6 (2.4–2.8) versus 2.1 (1.5–2.8) ( Table 3 ). Conversely for thromboembolic event–related hospital admissions, point estimates of the incidence rates were slightly lower during anticoagulant exposed than unexposed person-time, with rates of 0.2 (0.1–0.2) versus 0.4 (0.2–0.7) for VTE, and 3.1 (2.9–3.3) versus 3.3 (2.6–4.2) for the composite of ATE, respectively. Event rates were slightly higher in patients with cancer, but remained comparable regardless of anticoagulant use ( Table 4 ). One year after index, the cumulative incidences (95%CI), irrespective of anticoagulant exposure, were 2.4% (2.1–2.6) for bleeding, 0.2% (0.1–0.3) for VTE, and 2.9% (2.6–3.1) for ATE ( Table 3 ). Patients with cancer had a higher incidence of VTE, whereas patients with non-cancer diseases had a higher incidence of ATE ( Table 4 and Fig. 4 ).

**Table 3:** Cumulative incidences and incidence rates of first bleeding and thromboembolic events

	Events, *N*	IR/100 PY AC exposed (95%CI)	IR/100 PY unexposed (95%CI)	6-month cumulative incidence % (95%CI)	1-year cumulative incidence % (95%CI)	3-year cumulative incidence % (95%CI)
**Major and clinically relevant bleeding**	824	2.6 (2.4–2.8)	2.1 (1.5–2.8)	1.5 (1.3–1.7)	2.4 (2.1–2.6)	4.5 (4.2–4.8)
**Venous thromboembolism**	62	0.2 (0.1–0.2)	0.4 (0.2–0.7)	0.1 (0.1–0.2)	0.2 (0.1–0.3)	0.3 (0.2–0.4)
**Arterial thromboembolism**	983	3.1 (2.9–3.3)	3.3 (2.6–4.2)	1.8 (1.6–2.0)	2.9 (2.6–3.1)	5.5 (5.1–5.8)
** Myocardial infarction**	365	1.1 (1.0–1.3)	0.9 (0.5–1.4)	0.7 (0.6–0.9)	1.1 (1.0–1.3)	2.1 (1.9–2.3)
** Stroke**	520	1.6 (1.4–1.7)	2.0 (1.5–2.7)	0.9 (0.8–1.0)	1.4 (1.2–1.6)	2.8 (2.5–3.1)
** Other**	144	0.4 (0.4–0.5)	0.5 (0.2–0.9)	0.2 (0.2–0.3)	0.4 (0.3–0.5)	0.8 (0.7–1.0)

Abbreviations: AC, anticoagulation; CI, confidence interval; IR, incidence rate; PY, person-years.

Note: Cumulative incidences were computed taking the competing risk of death into account. Crude incidence rates (IR) of first bleeding and thromboembolic events were estimated as events per 100 person-years (PY), stratified by anticoagulant exposure.

**Table 4:** Cumulative incidences and incidence rates of first bleeding and thromboembolic events stratified by cancer versus non-cancer diseases

	Cancer					Non-cancer diseases				
	IR/100 PY AC exposed (95%CI)	IR/100 PY unexposed (95%CI)	6-month cumulative incidence % (95%CI)	1-year cumulative incidence % (95%CI)	3-year cumulative incidence % (95%CI)	IR/100 PY AC exposed (95%CI)	IR/100 PY unexposed (95%CI)	6-month cumulative incidence % (95%CI)	1-year cumulative incidence % (95%CI)	3-year cumulative incidence % (95%CI)
**Major and clinically relevant bleeding**	4.5 (3.6–5.7)	4.5 (2.1–8.6)	2.0 (1.5–2.7)	2.6 (2.0–3.3)	3.5 (2.8–4.3)	2.5 (2.3–2.7)	1.8 (1.3–2.5)	1.4 (1.2–1.6)	2.3 (2.1–2.6)	4.7 (4.3–5.0)
**Venous thromboembolism**	0.8 (0.5–1.4)	0.5 (0.0–2.5)	0.5 (0.3–0.9)	0.6 (0.4–1.0)	0.7 (0.4–1.1)	0.1 (0.1–0.2)	0.4 (0.2–0.8)	0.1 (0.0–0.1)	0.1 (0.1–0.2)	0.3 (0.2–0.4)
**Arterial thromboembolism**	3.0 (2.2–3.9)	7.1 (4.0–11.7)	1.7 (1.3–2.3)	2.1 (1.6–2.8)	2.9 (2.3–3.7)	3.1 (2.9–3.3)	2.9 (2.2–3.7)	1.9 (1.6–2.1)	3.0 (2.7–3.2)	5.9 (5.5–6.3)
** Myocardial infarction**	0.7 (0.3–1.2)	2.8 (1.0–6.0)	0.4 (0.2–0.8)	0.5 (0.3–0.9)	0.8 (0.5–1.2)	1.2 (1.0–1.3)	0.7 (0.4–1.2)	0.8 (0.7–0.9)	1.2 (1.0–1.4)	2.3 (2.0–2.5)
** Stroke**	1.8 (1.2–2.5)	3.7 (1.6–7.3)	1.0 (0.7–1.4)	1.3 (0.9–1.8)	1.7 (1.2–2.3)	1.6 (1.4–1.7)	1.8 (1.3–2.5)	0.9 (0.7–1.0)	1.4 (1.2–1.6)	3.0 (2.7–3.3)
** Other**	0.5 (0.2–0.9)	0.9 (0.1–3.3)	0.4 (0.2–0.7)	0.4 (0.2–0.7)	0.5 (0.2–0.8)	0.4 (0.4–0.5)	0.4 (0.2–0.8)	0.2 (0.2–0.3)	0.4 (0.3–0.5)	0.9 (0.7–1.0)

Abbreviations: AC, anticoagulation; CI, confidence interval; IR, incidence rate; PY, person-years.

Note: Cumulative incidences were computed taking the competing risk of death into account. Crude incidence rates (IR) of first bleeding and thromboembolic events were estimated as events per 100 person-years (PY), stratified by anticoagulant exposure.

### Sensitivity Analyses

Changing the construction of treatment periods based on anticoagulant prescriptions did not affect the observed rates of VKA or anticoagulant treatment discontinuation nor rates of bleeding or thromboembolic events stratified by anticoagulant exposure ( Supplementary Table S13 – S15 , available in the online version). Event rates were also comparable to our main analysis after extending the anticoagulant exposed period by 7 days ( Supplementary Table S14 , available in the online version).

## Discussion

Our large cohort study described the use and discontinuation of both VKAs and anticoagulant treatment overall and the incidence of bleeding and thromboembolic events in VKA users with a life-limiting disease. A key finding was the large proportion of prevalent VKA users who continued anticoagulant therapy during their last phase of life, with 69% remaining exposed to anticoagulants until death, and more than 80% continuing therapy until shortly before death. The observed VKA and anticoagulation discontinuation rates were relatively low at 17.9 for VKAs and 12.9 per 100 PYs for anticoagulants overall. Nevertheless, the incidence rates of hospital admission for bleeding and thromboembolic events were comparable during anticoagulant use and after discontinuation.

Discontinuation was more frequent in patients with cancer than in patients with non-cancer diseases, both for VKA and anticoagulant treatment overall. This difference may relate to the worse prognosis of cancer patients in our cohort, as illustrated by their median survival time of 0.35 years. Additionally, the bleeding risk associated with anticoagulant treatment may be higher in cancer than non-cancer patients, 43 leading physicians or patients to consider discontinuation sooner. Furthermore, the life expectancy in patients with cancer may be more predictable than that of patients with other life-limiting diseases, 44 although the introduction of immunotherapy and targeted therapy may have changed this dynamic over the last decade. 45 More frequent discontinuation of VKAs in cancer patients may also reflect clinical guidelines recommending LMWH or DOACs for treatment of cancer-associated thrombosis. 46

Our observation that a substantial proportion of VKA users continued anticoagulant therapy aligns with previous studies on anticoagulant use in patients with a life-limiting disease. 15 24 47 48 A chart review conducted in the Netherlands also demonstrated that anticoagulants were frequently continued until the last week(s) before death. Discontinuation of anticoagulation primarily occurred in response to a bleeding event, difficulty swallowing pills, or upon patient's request. 14 Several complex interacting factors may hinder deprescribing of anticoagulants during the last phase of life. One factor is prescribing inertia, defined as “ *the failure to act despite awareness that prescribing is potentially inappropriate.* ” 12 49 Prescribing inertia may arise from physicians', patients', or other stakeholders' fear of unknown or negative consequences of deprescribing. 12 Furthermore, the belief that the decision to continue or cease medication is the responsibility of another party (e.g., another prescriber, healthcare professional, or the patient) can also contribute to prescribing inertia. 11 12 Other barriers to deprescribing are the lack of awareness among prescribers and lack of evidence regarding the appropriateness of continuing anticoagulant use 12 and external factors, such as time constraints during patient encounters and guidelines that focus on prescribing rather than deprescribing. 11 12

Previous studies also observed comparable rates of hospital admissions and emergency department visits for thromboembolic and bleeding events during anticoagulant exposure and after discontinuation. 50 In patients with AF and active cancer, bleeding rates per 100 PYs (95%CI) were 7.2 (5.7–8.9) during anticoagulant treatment and 6.7 (2.1–16) after discontinuation and rates of thromboembolic complications were 5.4 (4.1–6.9) and 6.8 (2.2–16), respectively. 51 Among older (>65 years of age) recipients of home palliative care, incidence rates per 100 PYs (95%CI) were 12.7 (11.8–13.7) and 10.4 (8.7–12.3) for bleeding and 4.9 (4.3–5.5) and 5.2 (4.1–6.6) for thrombotic events during anticoagulant treatment versus after discontinuation, respectively. 15 In our study, the comparable event rates could be partly attributed to confounding by indication (i.e., the decision to continue or discontinue anticoagulants is likely influenced by the estimated risk of both bleeding and thromboembolism of the individual patient) and restricting to first events only. Therefore, these findings should not be interpreted in a causal way.

The magnitude of the event rates per 100 PYs in our cohort of VKA users with a life-limiting disease were comparable to those reported among the general population of VKA users in the Netherlands from 2013 to 2019, ranging between 1.3 to 3.0 for major bleeding and 0.75 to 0.85 for the composite of thromboembolic events. 52 As these events were based on interviews regularly performed during visits at the anticoagulation clinic and information provided by hospitals rather than hospital admissions only, event rates in patients with a life-limiting disease are likely higher than in the general population of VKA users. Furthermore, we observed higher event rates than those reported among newly diagnosed non-valvular AF patients treated with VKAs between 2010 and 2015: 1.27 major bleeding (95%CI 1.07–1.52) and 1.13 stroke and systemic embolism (95%CI 1.07–1.52) per 100 PYs. 53 Another study performed in a cohort of VKA users treated between 2009 and 2012 at an anticoagulation clinic in Groningen, the Netherlands, reported event rates stratified by age groups among patients aged 70 years or older. 54 Importantly, events were identified by computerized records from the clinic itself, complemented by information from general practitioners. The reported rates of clinically relevant non-major and major bleeding per 100 PYs were 14.8 in patients aged 70 to 79 years, 16.7 in patients aged 80 to 89 years, and 18.1 in patients 90 years or older. 54 For the composite of thrombotic events these were 0.8, 1.5, and 1.8, respectively. 54 The differences in event rates compared with our cohort may be attributed to the underlying risks of bleeding and thromboembolism in the population studied and differences in recording and identification of outcome events.

Strengths of our study include the large cohort of patients with limited life expectancy and different life-limiting diseases. In addition, we included both phenprocoumon and acenocoumarol users. Contrary to most other countries where warfarin is the most frequent VKA type, phenprocoumon and acenocoumarol are primarily used in the Netherlands. 55

Despite these strengths, several limitations of our study should be considered. First, misclassification and measurement error are inherent to the use of routinely collected healthcare data, especially for patients in the last phase of life who may not always be referred to a hospital. Moreover, we only had access to hospital admission data and lacked information on diagnoses made in outpatient settings, such as in nursing homes, hospices, and by general practitioners. Hence, our estimates likely underestimate the true event rate in this patient population. However, it should be noted that the DHD registry includes information on emergency room visits over 4 hours.

Second, we cannot entirely rule out the possibility of misclassification in the assessment of VKA, anticoagulant exposure, and treatment discontinuation. Although we limited misclassification by directly obtaining data on VKA treatment from anticoagulation clinics, not all Dutch anticoagulation clinics participated, and we lacked data on VKA treatment during hospital stay and in nursing homes. Additionally, anticoagulation clinics do not provide longitudinal data on anticoagulant exposure after switching to non-VKA anticoagulant therapies, such as DOACs or LMWHs. To further minimize misclassification, we used data on outpatient dispensed anticoagulant prescriptions to construct anticoagulant treatment periods over time after the registered VKA end date from the anticoagulation clinics. However, the prescription database lacked detail on quantity and dosage for collected prescriptions, requiring us to make assumptions about treatment duration. Furthermore, we were unable to distinguish therapeutic from prophylactic LMWH prescriptions. Reassuringly, our sensitivity analyses assessing these assumptions had little impact on our results.

Finally, we lacked information on whether patients received palliative care. An approach to study patients in their last phase of life would be to start follow-up when patients start receiving palliative care or are declared terminally ill. 48 Nevertheless, the validity and suitability of this method also depend on a homogenous definition of a palliative care patient 56 and the remaining follow-up time after being identified as such. Instead, we used a proxy by selecting patients with pre-specified life-limiting diseases and cancer types with a median survival of 3 years or less. This approach avoided selecting patients based on a future event (i.e., death), thereby decreasing the likelihood of selection bias in our study. However, presumably not all patients in our cohort truly had a life-limiting disease, at least not in its final stages.

## Conclusion

In conclusion, the majority of VKA users with life-limiting diseases continued anticoagulant therapy during their last phase of life. Among those who discontinued anticoagulant therapy, discontinuation typically occurred only shortly before death, and incidence rates of hospital admission for bleeding and thromboembolic events were similar during anticoagulant use and after discontinuation. Our findings indicate that actively deprescribing anticoagulants is uncommon. Further research is warranted to examine the risks and benefits of continuing versus discontinuing anticoagulants in patients with life-limiting diseases. Moreover, healthcare providers need evidence-based tools to support the process of shared decision-making about the use of anticoagulants during the last phase of life with patients and their caregivers. The SERENITY consortium is working toward developing and evaluating a shared decision-making support tool for this decision. 25

## Notes

**What is known about this topic?**

- In patients with a life-limiting disease, use of long-term preventive medications, including anticoagulants, should be reconsidered and drugs should be deprescribed if potential harms outweigh benefits.
- The limited amount of data on the risks of anticoagulant-associated bleeding and thromboembolism after discontinuing anticoagulants in these patients may create uncertainty for physicians and patients, complicating deprescribing decisions.

**What does this paper add?**

- This large-scale cohort study examined the current practice patterns of vitamin K antagonist (VKA) and overall anticoagulant treatment use in long-term VKA users with a life-limiting disease and showed that over 80% of patients continued anticoagulant therapy until shortly before death.
- Although incidence rates of hospital admission for bleeding and thromboembolic events were comparable during anticoagulant use and after discontinuation, these findings should not be interpreted in a causal way.
