# Supplementary material for: Discontinuation of Anticoagulants and Occurrence of Bleeding and Thromboembolic Events in Vitamin K Antagonist Users with a Life-Limiting Disease
Source: Thromb Haemost. 2025 Apr 4;126(7):714–28. doi: 10.1055/a-2524-5334 (PMC13286098; doi:10.1055/a-2524-5334)
Supplement: Supplementary file 1 — Supplementary Material [file 10-1055-a-2524-5334_28983125.pdf]

Supplementary Methods

Data Sources

Data on personal characteristics: This dataset provides data on personal characteristics (i.e., year of birth, sex, and immigration background) collected from the Personal Records Database (in Dutch "Basisregistratie Personen," BRP).[1] It includes all persons who have been registered in the BRP since October 1994, both residents (i.e., individuals who were registered in the population register of a Dutch municipality) and non-residents (i.e., individuals who had a relationship with the Dutch government).[2] In the Netherlands, it is compulsory for individuals who would stay in the Netherlands for more than 4 months to register at a Dutch municipality and therefore their demographic characteristics would be recorded in the BRP. For the current study, data from the calendar years 2013 to 2019 were used.

Data on household income: Data on household income were collected from the Tax and Customs administration and the student grant registration of the Education Executive Agency (in Dutch "Dienst Uitvoering Onderwijs," DUO).[2,3]

Data on mortality and causes of death: Mortality data includes information on the dates of death for all persons who have been registered in the BRP since October 1994.[4] Data on underlying causes of death of persons who were registered in the BRP and died since the 2013 statistical year were collected from the nationwide Dutch Registry of Causes of Death statistics.[5] This registry also contains information on the most important injury and the location of the accident for those who died of non-natural death. For all deceased persons, the location of death and the statistical year, or the year in which the deceased was included in the statistics, are also present.

Data on diagnoses registered during hospitalizations: Data on diagnoses registered at hospital admissions in Dutch hospitals were collected from the Dutch Hospital Data registry, which includes all general and academic Dutch hospitals and two short-stay hospital categories (i.e., a cancer clinic and an eye hospital)[2] and includes data from individuals registered in the BRP.[6–9] The data contain diagnoses retrieved from discharge letters, length of hospital stay, and date of admission/discharge. The variable main diagnosis indicated which diagnosis was the main reason for the corresponding hospital admission, and the variable primary diagnosis indicated whether a diagnosis was the main reason for providing the corresponding care.[10]

Data on outpatient medication prescriptions: Data on outpatient medication prescriptions for which costs were reimbursed under the basic health insurance in the Netherlands. The data included information on medications for individuals in residential homes for elderly, whereas information on medications in hospitals and in nursing homes were not included.[10,11] Only the prescription year was available.

Data on outpatient medication prescriptions of anticoagulants: Data on outpatient dispensed prescriptions of anticoagulants, identified by the codes from the World Health Organization's Anatomic-Therapeutic-Chemical (ATC) system (i.e., B01A*). Data contain information on the type of anticoagulant and the dispensing date, but information on the amount of medication per dispensed prescription and the anticoagulant subtypes were not available.[10]

Data from Dutch anticoagulation clinics: These data comprise information about VKA treatments managed by participating anticoagulation clinics, including information on start date, indication(s) for treatment, type of VKAs, dose, target INR ranges, INR values and measurement time, reason for stop, and stop date (if available). These clinics are managed by the Dutch Federation of Anticoagulation Clinics (in Dutch "Federatie Nederlandse Trombosediensten," FNT). The following anticoagulation clinics participated in the study: Leiden Anticoagulation Clinic (Leiden), Atalmedial (Amsterdam), Saltro (Utrecht), Star-shl (Rotterdam), and Isala (Zwolle).

Netherlands Cancer Registry (NCR): The NCR is provided by the Netherlands Comprehensive Cancer Organization (in Dutch "Integraal Kankercentrum Nederland," IKNL) and comprises individual-level data of newly diagnosed patients with cancer in the Netherlands, including cancer diagnosis, tumor staging (according to the TNM classification developed and maintained by the Union for International Cancer Control [UICC]), tumor site (topography) and morphology (histology) (according to the WHO International Classification of Diseases for Oncology [ICD-O-3]), comorbidity at diagnosis, and treatment received directly after diagnosis (source: Netherlands Cancer Registry [NCR] (iknl.nl)).

Data Linkage

Data from the anticoagulation clinics were linked to data from the Statistics Netherlands by sex, date of birth, postal code, and last date known to be alive and >95% of records were successfully matched. Data from the NCR were also linked to the Statistics Netherlands by sex, date of birth, and postal code with a 98.5% match.

Details about Constructing Treatment Periods for Anticoagulants

Dispensed anticoagulant prescriptions for vitamin K antagonists (VKAs), direct oral anticoagulants (DOACs), and low-molecular-weight heparins (LMWHs) from 2012 to 2020 were used to examine VKA and anticoagulant exposure after registered VKA end dates from the anticoagulation clinics, to account for possible switching to non-VKA anticoagulants and non-participating anticoagulation clinics. Exposure to both VKA and anticoagulant was modeled by constructing treatment periods of person–time exposed according to dispensed prescriptions, only considering prescriptions after VKA end dates

 © 2025. The Author(s).

from anticoagulation clinics. As no data were available on the amount of anticoagulant dispensed nor the prescribed dose, treatment periods for the different types of anticoagulants were constructed assuming that a dispensing lasted a fixed number of days (i.e., the exposure time) unless a refill, death, or end of follow-up occurred earlier. Whenever the period between two subsequent dispensed prescription dates exceeded this fixed number of days, the next prescription was considered to belong to a new treatment period. A predefined number of days was added to the last prescription date within the treatment period to construct the end of the corresponding treatment period (►**Supplementary Fig. S1**). For VKAs, the exposure time of a single prescription was based on a previously performed validation study (Kempers et al, 2025, unpublished data), which resulted in 180 days allowed between subsequent VKA prescription dates and the addition of 60 days to the last prescription date to construct the end date of the corresponding treatment period. For DOACs and LMWHs, exposure times were based on the number of days between two dispensed prescriptions studied in a random sample of patients with DOAC and/or LMWH prescriptions between 2013 and 2019 (►**Supplementary Fig. S2**). We applied an exposure time of 120 days for DOAC prescriptions and 30 days for LMWH prescriptions.

As sensitivity analyses, we varied the exposure time of anticoagulant prescriptions when constructing treatment periods: 100 days for LMWH prescriptions, 150 days combined with the addition of 100 days to the last prescription date within the treatment period for VKA prescriptions.

R Packages Used

The following packages were used for statistical analyses and preparing figures: tidyverse,[12] dplyr,[13] lubridate,[14] stringr,[15] tidyr,[16] foreign,[17] forcats,[18] purrr,[19] ggplot2,[20] survival,[21] prodlim,[22] epiR,[23] tidycmprks,[24] ggsurvfit,[25] and cowplot.[26]

 © 2025. The Author(s).

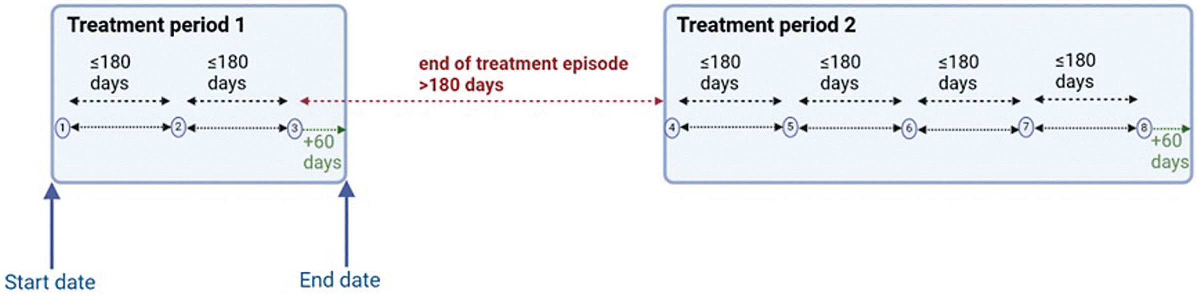

Supplementary Fig. S1 Construction of treatment periods for anticoagulants. Treatment periods for anticoagulants were constructed assuming that a dispensed prescription lasted a fixed number of days, unless a refill, death, or end of follow-up occurred earlier. Whenever the period between two subsequent dispensed prescription dates exceeded this fixed number of days, the next prescription was considered to belong to a new treatment period. A predefined number of days was added to the last prescription date within the treatment period to construct the end of the corresponding treatment period. Different exposure times were applied to the different types of anticoagulants: 180 days for VKA prescriptions, 120 days for DOAC prescriptions, and 30 days for LMWH prescriptions. The end date of each treatment period was constructed by adding a fixed number of days to the last dispensing date within the treatment period. This was 60 days for VKA prescriptions, 120 days for DOAC prescriptions, and 30 days for LMWH prescriptions. (Created in BioRender. Kruip, M. (2024) https://BioRender.com/y81a190.)

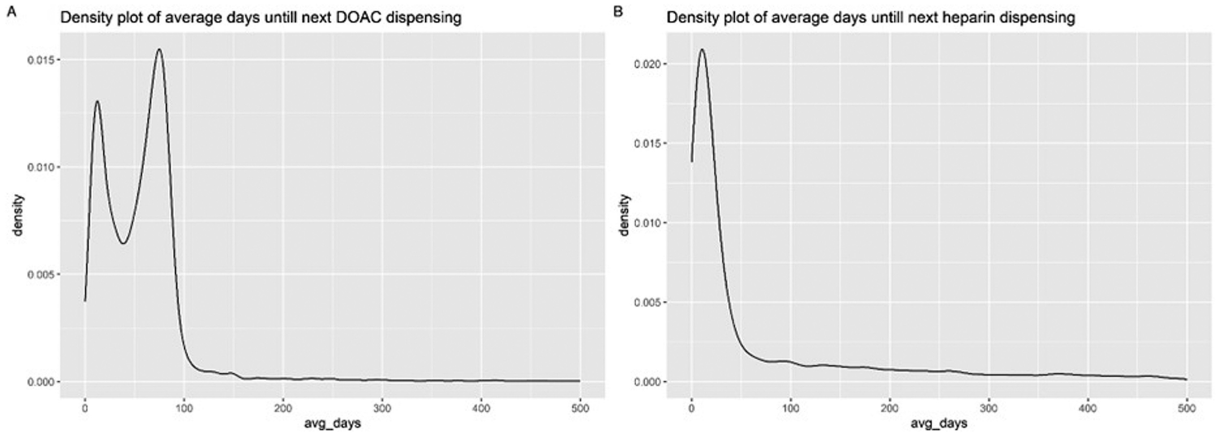

Supplementary Fig. S2 Density plots of average days until next DOAC or heparin dispensed prescription. Density plots of average days until next DOAC **(A)** or heparin **(B)** dispensation per patient studied in two random samples of 10,000 patients with DOAC or heparin prescriptions between 2013 and 2019, respectively. For patients with DOAC prescriptions, the median number of days until the next DOAC dispensation per patient was 58 days with a 95th percentile of 117 days. For heparin the median was 22 days.

 © 2025. The Author(s).

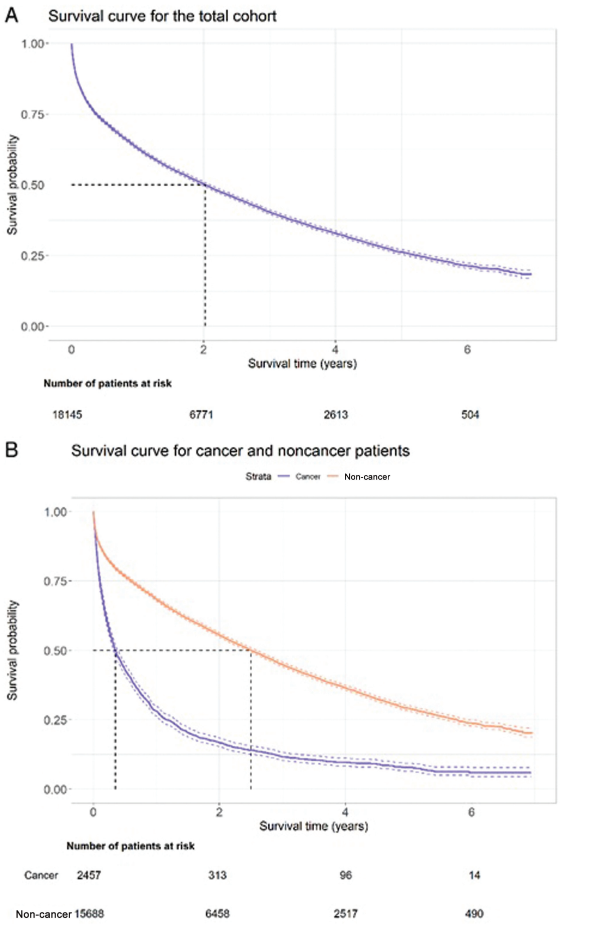

Supplementary Fig. S3 Kaplan-Meier survival curve for the total cohort and for patients with cancer versus non-cancer diseases. Kaplan-Meier survival curves for the total cohort (**A**) and patients with cancer versus non-cancer diseases separately (**B**). Median survival was estimated by the Kaplan-Meier estimator.

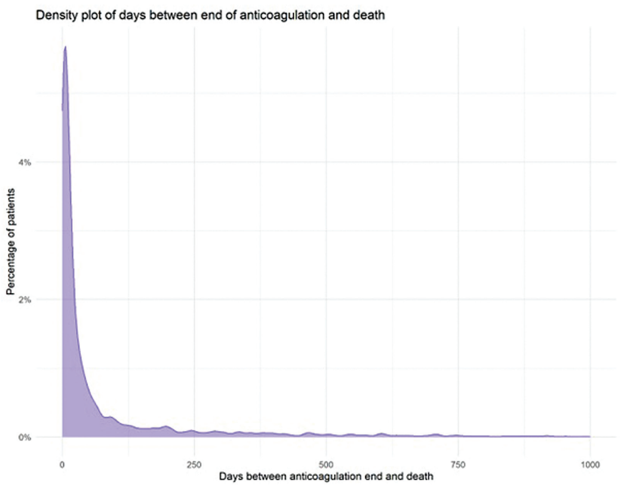

Supplementary Fig. S5 Density plot of days between end of anticoagulant treatment and death. Calculated among patients who died during follow-up and discontinued anticoagulant treatment before death ($N = 3{,}372$).

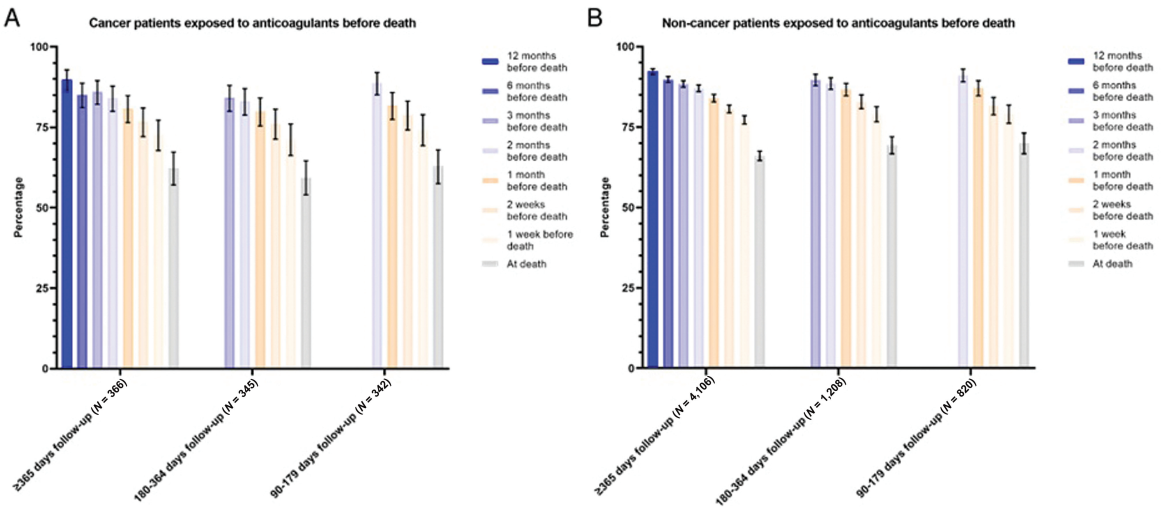

Supplementary Fig. S4 Patients exposed to anticoagulants before death stratified by cancer versus non-cancer diseases. Percentage of patients exposed to anticoagulants with corresponding 95% confidence intervals at different time points before death, stratified according to the amount of follow-up time between index date and date of death and by cancer versus non-cancer diseases. This analysis was restricted to patients who died during follow-up.

Supplementary Table S1 ICD-10 codes used to identify non-cancer life-limiting diseases

Life-limiting disease	Restrictions	ICD-10 codes
**Liver disease**		
Esophageal varices	Primary diagnosis of hospital admission	I85.0
Alcoholic cirrhosis of liver	Primary diagnosis of hospital admission	K70.3
Secondary biliary cirrhosis	Primary diagnosis of hospital admission	K74.4
Unspecified cirrhosis of liver	Primary diagnosis of hospital admission	K74.6
Hepatorenal syndrome	Primary diagnosis of hospital admission	K76.7
**Hip fracture**		
Fracture of neck of femur	Primary diagnosis of hospital admission in patients >70 years	S72.0
Pertrochanteric fracture	Primary diagnosis of hospital admission in patients >70 years	S72.1
Subtrochanteric fracture	Primary diagnosis of hospital admission in patients >70 years	S72.2
**Heart disease**		
Hypertensive heart disease with heart failure	Primary diagnosis of hospital admission	I11.0
Hypertensive heart and chronic kidney disease with heart failure and stage 1 through stage 4 chronic kidney disease, or unspecified chronic kidney disease	Primary diagnosis of hospital admission	I13.0
Hypertensive heart and chronic kidney disease with heart failure and with stage 5 chronic kidney disease, or end-stage renal disease	Primary diagnosis of hospital admission	I13.2
Ischemic cardiomyopathy	Primary diagnosis of hospital admission	I25.5
Heart failure	Primary diagnosis of hospital admission	I50.1; I50.2; I50.3; I50.4; I50.8; I50.9
**Lung disease**		
Mixed simple and mucopurulent chronic bronchitis	Primary diagnosis of hospital admission	J41.8
Other chronic obstructive pulmonary disease	Primary diagnosis of hospital admission	J440; J441; J449
Other interstitial pulmonary diseases with fibrosis	Primary diagnosis of hospital admission	J84.1
**Diabetes mellitus**		
Type 1 diabetes mellitus with kidney complications	Primary diagnosis of hospital admission in combination with diagnosis of severity (see ►**Supplementary Table S2**)	E10.2
Type 1 diabetes mellitus with circulatory complications	Primary diagnosis of hospital admission in combination with diagnosis of severity	E10.5
Type 1 diabetes mellitus with multiple complications	Primary diagnosis of hospital admission in combination with diagnosis of severity	E10.7
Type 2 diabetes mellitus with kidney complications	Primary diagnosis of hospital admission in combination with diagnosis of severity	E11.2
Type 2 diabetes mellitus with circulatory complications	Primary diagnosis of hospital admission in combination with diagnosis of severity	E11.5
Type 2 diabetes mellitus with multiple complications	Primary diagnosis of hospital admission in combination with diagnosis of severity	E11.7
Malnutrition-related diabetes mellitus with renal complications	Primary diagnosis of hospital admission in combination with diagnosis of severity	E12.2
Malnutrition-related diabetes mellitus with peripheral circulatory complications	Primary diagnosis of hospital admission in combination with diagnosis of severity	E12.5
Malnutrition-related diabetes mellitus with multiple complications	Primary diagnosis of hospital admission in combination with diagnosis of severity	E12.7

 © 2025. The Author(s).

Supplementary Table S1 (*Continued*)

Life-limiting disease	Restrictions	ICD-10 codes
Other specified diabetes mellitus with kidney complications	Primary diagnosis of hospital admission in combination with diagnosis of severity	E13.2
Other specified diabetes mellitus with circulatory complications	Primary diagnosis of hospital admission in combination with diagnosis of severity	E13.5
Other specified diabetes mellitus with multiple complications	Primary diagnosis of hospital admission in combination with diagnosis of severity	E13.7
Unspecified diabetes mellitus with renal complication	Primary diagnosis of hospital admission in combination with diagnosis of severity	E14.2
Unspecified diabetes mellitus with peripheral circulatory complications	Primary diagnosis of hospital admission in combination with diagnosis of severity	E14.5
Unspecified diabetes mellitus with multiple complications	Primary diagnosis of hospital admission in combination with diagnosis of severity	E14.7
**Dementia**		
Creutzfeldt-Jakob disease	Primary or secondary diagnosis of hospital admission	A81.0
Subacute sclerosing panencephalitis	Primary or secondary diagnosis of hospital admission	A81.1
Progressive multifocal leukoencephalopathy	Primary or secondary diagnosis of hospital admission	A81.2
Other atypical virus infections of central nervous system	Primary or secondary diagnosis of hospital admission	A81.8
Vascular dementia, unspecified severity	Primary or secondary diagnosis of hospital admission	F01.5
Vascular dementia, severe	Primary or secondary diagnosis of hospital admission	F01.C
Dementia in other diseases classified	Primary or secondary diagnosis of hospital admission	F02.8
Dementia in other diseases classified, severe	Primary or secondary diagnosis of hospital admission	F02.C
Unspecified dementia, unspecified severity	Primary or secondary diagnosis of hospital admission	F03.9
Unspecified dementia, severe	Primary or secondary diagnosis of hospital admission	F03.C
Delirium superimposed on dementia	Primary or secondary diagnosis of hospital admission	F05.1
Alzheimer's disease	Primary or secondary diagnosis of hospital admission	G30
Frontotemporal dementia	Primary or secondary diagnosis of hospital admission	G31.0
Senile degeneration of brain	Primary or secondary diagnosis of hospital admission	G31.1

 © 2025. The Author(s).

Supplementary Table S2 ICD-10 codes used to identify any diagnosis of severity to indicate the severity of diabetes mellitus

Any diagnosis of severity in combination with diabetes mellitus	ICD-10 codes
**Peripheral vascular disease**	
Diabetes mellitus due to underlying condition with circulatory complications	E08.5
Drug- or chemical-induced diabetes mellitus with circulatory complications	E09.5
Atherosclerosis of unspecified type of bypass graft(s) of the extremities	I70.3
Atherosclerosis of autologous vein bypass graft(s) of the extremities	I70.4
Atherosclerosis of nonautologous biological bypass graft(s) of the extremities	I70.5
Atherosclerosis of nonbiological bypass graft(s) of the extremities	I70.6
Atherosclerosis of other type of bypass graft(s) of the extremities	I70.7
Other and unspecified atherosclerosis	I70.9
Other arterial dissection	I77.7
**Coronary artery disease**	
Angina	I20.0; I20.1; I20.8; I20.9
ST elevation (STEMI) myocardial infarction involving other sites	I21.0; I21.1; I21.2; I21.3; I21.4; I21.9
Subsequent ST elevation (STEMI) and non-ST elevation (NSTEMI) myocardial infarction	I22.0; I22.1; I22.8; I22.9
Other acute ischemic heart diseases	I24.0; I24.1; I24.8; I24.9
Atherosclerotic heart disease of native coronary artery	I25.1
Old myocardial infarction	I25.2
Coronary artery aneurysm and dissection	I25.4
Ischemic cardiomyopathy	I25.5
Silent myocardial ischemia	I25.6
Atherosclerosis of coronary artery bypass graft(s) and coronary artery of transplanted heart with angina pectoris	I25.7
Atherosclerosis of other coronary vessels without angina pectoris	I25.8
Chronic ischemic heart disease, unspecified	I25.9
**Kidney failure**	
Hypertensive renal disease with renal failure	I12.0
Hypertensive heart and chronic kidney disease without heart failure	I13.1
Chronic kidney disease, stage 4	N18.4
Chronic kidney disease, stage 5	N18.5
End-stage renal disease	N18.6
Unspecified kidney failure	N19
Encounter for adequacy testing for hemodialysis	Z49.3
Dependence on renal dialysis	Z99.2

 © 2025. The Author(s).

Supplementary Table S3 Survival rates of different cancer diagnoses

Cancer location	Stage	1-year survival	3-year survival	5-year survival	Reference
**Pancreas**	All stages	22%	6%	<5%	van der Geest L, et al. Alvleesklier in Nederland, kleine stappen vooruit. IKNL; 2021.
Cervix					
	IIA2-IVA (locally advanced)	86%	66%	59%	van der Aa M, et al. (no date) Baarmoederhalskanker in Nederland. Accessed September 17, 2024) at: https://iknl.nl/cervixcarcinoom-in-nederland.
	IIIB	71%	42%	37%	
	IVA	47%	24%	21%	
	IVB	38%	13%	7%	
Endometrium[a]		90%	83%	80%	Soslow RA, et al. Clinicopathologic analysis of 187 high-grade endometrial carcinomas of different histologic subtypes: similar outcomes belie distinctive biologic differences. The American Journal of Surgical Pathology. 2007;31(7):979–987.
	III	80%		48%	
	IV	48%		15%	
Bladder					
	T2–4a			45.7%	Ripoll J, et al. Cancer-specific survival by stage of bladder cancer and factors collected by Mallorca Cancer Registry associated to survival. BMC Cancer. 2021;21(1):676.
	T3	60%	42%	41.0%	
	T4a + b	IV: 40%	IV: 20%	21%; 12%	
Breast					
	III	96%	83%	73%	IKNL. Overleving borstkanker. Published 2023. Accessed September 17, 2024 at: https://iknl.nl/kankersoorten/borstkanker/registratie/overleving.
	IV	70%	38%	22%	
Bone and soft tissue (sarcoma)					
	Bone	83%	69%	63%	IKNL. Overleving bot- en wekedelenkanker. Published 2024. Accessed September 17, 2024 at: https://iknl.nl/kankersoorten/bot-en-wekedelenkanker/registratie/overleving.
	Soft tissue sarcoma	79%	65%	59%	
	GIST	91%	84%	80%	
Colon					
	III	88–92%		71%	Araghi M, et al. Colon and rectal cancer survival in seven high-income countries 2010–2014: variation by age and stage at diagnosis (the ICBP SURVMARK-2 project). Gut. 2021;70(1):114–126.
	IV	40–58%		12%	
Rectal					
	III	91–96%		75%	Araghi M, et al. Colon and rectal cancer survival in seven high-income countries 2010–2014: variation by age and stage at diagnosis (the ICBP SURVMARK-2 project). Gut. 2021;70(1):114–126.
	IV	52–66%		14%	

(*Continued*)

 © 2025. The Author(s).

Supplementary Table S3 (*Continued*)

Cancer location	Stage	1-year survival	3-year survival	5-year survival	Reference
**Ovary**					
	III	80%	40%	25%	Gaitskell K, et al. Ovarian cancer survival by stage, histotype, and pre-diagnostic lifestyle factors, in the prospective UK Million Women Study. Cancer Epidemiology. 2022;76:102074.
	IV	75%	25%	12%	
**Brain**		54%	31%	26%	IKNL. Overleving Hersentumoren. Published 2023. Accessed September 17, 2024 at: https://iknl.nl/kankersoorten/hersentumoren/registratie/overleving.
**Hepatobiliary—liver**		44%	24%	18%	IKNL. Overleving HPB-tumoren. Published 2023. Accessed September 17, 2024 at: https://iknl.nl/kankersoorten/hpb-tumoren/registratie/overleving.
**Hepatobiliary—gallbladder/bile duct**		46%	22%	17%	IKNL. Overleving HPB-tumoren. Published 2023. Accessed September 17, 2024 at: https://iknl.nl/kankersoorten/hpb-tumoren/registratie/overleving.
**Lung**			34%		IKNL. Overleving longkanker. Published 2024. Accessed September 17, 2024 at: https://iknl.nl/kankersoorten/longkanker/registratie/overleving.
	NSCLC III	78%	42%	30%	
	NSCLC IV	33%	<10%	<5%	
	SCLC III	78%	35%	24%	
	SCLC IV	25%	<10%	<5%	
**NEC/NET (any location)**[b]	All stages	73%	53%	39%	Man D, et al. Prognosis of patients with neuroendocrine tumor: a SEER database analysis. Cancer Management and Research. 2018;10:5629–5638. Dasari A, et al. Trends in the incidence, prevalence, and survival outcomes in patients with neuroendocrine tumors in the United States. JAMA Oncology. 2017;3(10):1335–1342.
**Primary tumor unknown**					
	Treated	40%		20%	IKNL. Overleving Primaire Tumor Onbekend. Published 2024. Accessed September 17, 2024 at: https://iknl.nl/kankersoorten/primaire-tumor-onbekend/registratie/overleving.
	Untreated	5%	<5%	<5%	
**Esophagus**					
	III	65%	33%	26%	IKNL. Overleving slokdarm- en maagkanker. Published 2023. Accessed September 17, 2024 at: https://iknl.nl/kankersoorten/slokdarm-en-maagkanker/registratie/overleving.
	IV	22%	4%	2%	

 © 2025. The Author(s).

Supplementary Table S3 (*Continued*)

Cancer location	Stage	1-year survival	3-year survival	5-year survival	Reference
**Gastric**					
	III	60%	25%	16%	IKNL. Overleving slokdarm- en maagkanker. Published 2023. Accessed September 17, 2024 at: https://iknl.nl/kankersoorten/slok-darm-en-maagkanker/registratie/overleving.
	IV	17%	2%	1%	

Notes: First, all survival rates were collected by consulting the Web site of the Netherlands Comprehensive Cancer Organization ("IKNL" in Dutch). If 3- or 5-year survival rates were not available on the IKNL Web site, epidemiological literature was used to identify the survival rates. Cancer types and stages with a 3-year survival rate of 50% or less were included in the study.
[a]Good quality epidemiological studies describing 1-, 3-, or 5-year survival rates were found; hence, this more pathology focused paper was used for the survival rates.
[b]The 3-year survival rates for all NET/NECs in general were above 50%. However, as described in the paper from Dasari et al, grade 3 and 4 patients had a much lower survival rate than grade 1 and 2 patients. Looking at the overall 3-year survival rate of 53%, it was chosen to include the grade 3 and 4 patients but not the grade 1 and 2 patients.

Supplementary Table S4 Description of included severe cancer diagnoses

All stages	Stage III + IV	Stage IV
Pancreas	Cervix	Breast
Primary tumor unknown	Bladder	Colon and rectum
Brain	Ovary	Endometrium
Hepatobiliary (liver and gall bladder/duct)	Lung (NSCLC and SCLC)	
	Neuro-endocrine tumors	
	Esophagus	
	Gastric	

Note: Cancer types with a median survival time of 3 years or less at the time of first diagnosis were selected.

Supplementary Table S5 ICD codes used to identify comorbidities

Comorbidities	Type of code	Code(s)
Asthma	ICD-10	J45, J46
	ICD-9	493
Chronic obstructive pulmonary disease	ICD-10	J44
	ICD-9	491, 492, 496
Other chronic lung diseases	ICD-10	J41, J42, J43, J47, J6, J7
	ICD-9	494, 495, 50
Heart failure	ICD-10	I50
	ICD-9	428
Hypertension	ICD-10	I10, I11, I13, I15
	ICD-9	401, 402, 404, 405
Atrial fibrillation	ICD-10	I48
	ICD-9	4273
Atherosclerosis	ICD-10	I20, I250, I251, I255, I258, I259, I70
	ICD-9	413, 4140, 4143, 4144, 4148, 4149, 4292, 440
Myocardial infarction (history)	ICD-10	I21, I22, I252
	ICD-9	410, 412, 4142

(*Continued*)

 © 2025. The Author(s).

Supplementary Table S5 (*Continued*)

Comorbidities	Type of code	Code(s)
Rheumatic heart disease	ICD-10	I05, I06, I07
	ICD-9	3941, 395, 3971
Other valvular heart disease	ICD-10	I08, I34, I35, I36, I37, I38, I39, Z952
	ICD-9	3940, 3942, 3949, 396, 3970, 424, V433
Peripheral artery disease	ICD-10	I739
	ICD-9	4439
Liver disease	ICD-10	B15, B16, B17, B18, B19, D684, I982, I983, K70, K71, K72, K73, K74, K75, K76, K77, Z944
	ICD-9	070, 4560, 4561, 4562, 571, 5722, 5723, 5724, 5728, 5731, 5732, 5733, 5735, V427
Diabetes	ICD-10	E10, E11, E12, E13, E14
	ICD-9	250
Thyroid disease	ICD-10	E00, E01, E02, E03, E04, E05, E06, E07
	ICD-9	240, 241, 242, 243, 244, 245, 246
Kidney disease	ICD-10	I12, N01, N02, N03, N04, N05, N06, N07, N08, N11, N12, N14, N150, N158, N159, N16, N18, N19, N25, N26, Q60, Q611, Q612, Q613, Q614, Q615, Q618, Q619, Z49, Z940
	ICD-9	403, 581, 582, 583, 585, 586, 587, 588, 5900, 7530, 7531, V420, V451, V56
Anemia	ICD-10	D5, D60, D61, D63, D64
	ICD-9	280, 281, 282, 283, 2840, 2848, 2849, 2850, 2852, 2858, 2859
Coagulopathy	ICD-10	D65, D66, D67, D680, D681, D682, D683, D685, D686, D688, D689, D69
	ICD-9	286, 287
Stroke/TIA (history)	ICD-10	G45, G46, I63, I64
	ICD-9	3623, 434.1, 434.9, 435, 436
(Other) arterial thromboembolism	ICD-10	H340, H341, H342, I513, I74, K550
	ICD-9	3623, 444, 5570
Venous thromboembolism (history)	ICD-10	H348, I26, I636, I676, I801, I802, I803, I808, I809, I81, I820, I822, I823, I828, I829, K765, O225, O873
	ICD-9	4340, 4376, 4511, 4512, 4518, 4519, 452, 4530, 4532, 4533, 4534, 4536, 4538, 4539, 6715
Major and clinically relevant bleeding (history)	ICD-10	D62, D683, H356, H431, I230, I312, I60, I61, I62, I850, I983, J942, K226, K250, K252, K254, K256, K260, K262, K264, K266, K270, K272, K274, K276, K280, K282, K284, K286, K290, K625, K661, K920, K921, K922, M250, N02, N837, N920, N921, N924, N938, N939, N950, R04, R31, R58, S064, S065, S066
	ICD-9	2851, 2865, 4230, 430, 431, 432, 4560, 4590, 5307, 5310, 5312, 5314, 5316, 5320, 5322, 5324, 5326, 5330, 5332, 5334, 5336, 5340, 5342, 5344, 5346, 5350, 5693, 578, 5967, 5997, 6207, 6262, 6266, 6268, 6269, 6270, 6271, 7191, 7847, 7848, 7863, 852
Parkinson's disease	ICD-10	F023, G20
	ICD-9	3320
Alzheimer's disease	ICD-10	F00, G30
	ICD-9	3310

 © 2025. The Author(s).

Supplementary Table S5 (*Continued*)

Comorbidities	Type of code	Code(s)
Immune deficiency	ICD-10	D80, D81, D82, D83, D84, D89
	ICD-9	2790, 2791, 2792, 2793, 2798, 2799
Autoimmune/autoinflammatory disease	ICD-10	D86, E271, G35, K50, K51, K900, M05, M06, M07, M08, M09, M30, M31, M32, M33, M34, M35
	ICD-9	135, 2554, 2794, 340, 556, 5790, 6960, 710, 714, 725
Malignant tumor	ICD-10	C
	ICD-9	14, 15, 16, 17, 18, 19, 20

Supplementary Table S6 ATC codes used to identify medication prescription at the pharmacological subgroup level at index date

Pharmacological subgroup	ATC code
Antihypertensives	C02, C03, C07, C08, C09
Lipid-lowering drugs	C10
Nonsteroid anti-inflammatory and anti-rheumatic drugs	M01A
Steroids	H02A, H02B
Antidepressants	N06A
Antacids	A02B
Antiplatelet drugs	B01AC

Note: Data on outpatient medication prescriptions covered by the Dutch statutory basic medical insurance were obtained from Statistics Netherlands. These included the year of prescription and Anatomical Therapeutic Chemical (ATC) code. Medications received in hospitals and nursing homes were not available. Only for anticoagulants, more detailed prescription data were provided, including dispending date and type of anticoagulant. The latter were used to identify prescriptions of antiplatelet drugs.

 © 2025. The Author(s).

Supplementary Table S7 ICD-10 codes used to identify bleeding and thromboembolic events

<table>
<tr><th colspan="4">Major and clinically relevant bleeding</th></tr>
<tr>
<td colspan="2">D62: Acute posthemorrhagic anemia
D683: Hemorrhagic disorder due to circulating anticoagulants
H356: Retinal hemorrhage
H431: Vitreous hemorrhage
I230: Hemopericardium as current complication following acute myocardial infarction
I312: Hemopericardium, not elsewhere classified
I60: Subarachnoid hemorrhage
I61: Intracerebral hemorrhage
I62: Other nontraumatic intracranial hemorrhage
I850: Esophageal varices with bleeding
I983: Esophageal varices with bleeding in diseases classified elsewhere
J942: Hemothorax
K226: Gastro-esophageal laceration-hemorrhage syndrome
K250: Gastric ulcer, acute with hemorrhage
K252: Gastric ulcer, acute with both hemorrhage and perforation
K254: Gastric ulcer, chronic or unspecified with hemorrhage</td>
<td>K256: Gastric ulcer, chronic or unspecified with both hemorrhage and perforation
K260: Duodenal ulcer, acute with hemorrhage
K262: Duodenal ulcer, acute with both hemorrhage and perforation
K264: Duodenal ulcer, chronic or unspecified with hemorrhage
K266: Duodenal ulcer, chronic or unspecified with both hemorrhage and perforation
K270: Peptic ulcer, site unspecified, acute with hemorrhage
K272: Peptic ulcer, site unspecified, acute with both hemorrhage and perforation
K274: Peptic ulcer, site unspecified, chronic or unspecified with hemorrhage
K276: Peptic ulcer, site unspecified, chronic or unspecified with both hemorrhage and perforation
K280: Gastrojejunal ulcer, acute with hemorrhage
K282: Gastrojejunal ulcer, acute with both hemorrhage and perforation
K284: Gastrojejunal ulcer, chronic or unspecified with hemorrhage
K286: Gastrojejunal ulcer, chronic or unspecified with both hemorrhage and perforation
K290: Acute hemorrhagic gastritis
K625: Hemorrhage of anus and rectum
K661: Hemoperitoneum</td>
<td>K920: Hematemesis
K921: Melaena
K922: Gastrointestinal hemorrhage, unspecified
M250: Hemarthrosis
N02: Recurrent and persistent hematuria
N837: Hematoma of broad ligament
N920: Excessive and frequent menstruation with regular cycle
N921: Excessive and frequent menstruation with irregular cycle
N924: Excessive bleeding in the premenopausal period
N938: Other specified abnormal uterine and vaginal bleeding
N939: Abnormal uterine and vaginal bleeding, unspecified
N950: Postmenopausal bleeding
R04: Hemorrhage from respiratory passages
R31: Unspecified hematuria
R58: Hemorrhage, not elsewhere classified
S064: Epidural hemorrhage
S065: Traumatic subdural hemorrhage
S066: Traumatic subarachnoid hemorrhage</td>
</tr>
<tr><th colspan="4">Venous thromboembolism</th></tr>
<tr>
<td>PE/DVT + thrombophlebitis
I26: Pulmonary embolism
I801: Phlebitis and thrombophlebitis of femoral vein
I802: Phlebitis and thrombophlebitis of other deep vessels of lower extremities (Deep vein thrombosis NOS)
I803: Phlebitis and thrombophlebitis of lower extremities, unspecified (Embolism or thrombosis of lower extremity NOS)</td>
<td>VTE other
H348: Other retinal vascular occlusions
I808: Phlebitis and thrombophlebitis of other sites
I809: Phlebitis and thrombophlebitis of unspecified site
I820: Budd-Chiari
I821: Thrombophlebitis migrans
I822: Embolism and thrombosis of vena cava
I823: Embolism and thrombosis of renal vein
I828: Embolism and thrombosis of other specified veins</td>
<td>Cerebral sinus thrombosis
I636: Cerebral infarction due to cerebral venous thrombosis, nonpyogenic
I676: Nonpyogenic thrombosis of intracranial venous system
O225: Cerebral venous thrombosis in pregnancy
O873: Cerebral venous thrombosis in the puerperium</td>
<td>Portal vein thrombosis
I81: Portal vein thrombosis</td>
</tr>
</table>

 © 2025. The Author(s).

Supplementary Table S7 (*Continued*)

**Major and clinically relevant bleeding**			
	I829: Embolism and thrombosis of unspecified vein<br>K765: Hepatic veno-occlusive disease<br>O223: Deep phlebothrombosis in pregnancy (deep vein thrombosis, antepartum)<br>O229: Venous complication in pregnancy, unspecified<br>O871: Deep phlebothrombosis in the puerperium (deep vein thrombosis, postpartum/pelvic thrombophlebitis, postpartum)<br>O879: Venous complication in the puerperium, unspecified<br>O882: Obstetric blood-clot embolism (obstetric [pulmonary] embolism NOS/puerperal [pulmonary] embolism NOS)		
**Arterial thromboembolism**			
**Ischemic stroke/TIA**<br>I63: Cerebral infarction<br>G45: Transient cerebral ischemic attacks and related syndromes<br>G46: Vascular syndromes of brain in cerebrovascular diseases<br>I64: Stroke, not specified as hemorrhage or infarction		**Other arterial thromboembolism**<br>H340: Transient retinal artery occlusion<br>H341: Central retinal artery occlusion<br>H342: Other retinal artery occlusions<br>I513: Intracardial thrombosis<br>I74: Arterial embolism and thrombosis<br>K550: Acute vascular disorder of intestine	**Myocardial infarction**<br>I21: Acute myocardial infarction<br>I22: Subsequent myocardial infarction

 © 2025. The Author(s).

Supplementary Table S8 Additional baseline characteristics

	Total ($N = 18,145$)	Cancer ($N = 2,457$)	Non-cancer ($N = 15,688$)
**Standardized household income, quintile[a], No. (%)**			
First (lowest)	4,207 (23.2)	589 (24.0)	3,618 (23.1)
Second	6,401 (35.3)	787 (32.0)	5,614 (35.8)
Third	3,009 (16.6)	471 (19.2)	2,538 (16.2)
Fourth	1,923 (10.6)	295 (12.0)	1,628 (10.4)
Fifth (highest)	1,408 (7.8)	261 (10.6)	1,147 (7.3)
Institutional household/unknown	1,197 (6.6)	54 (2.2)	1,143 (7.3)
**≥1 comorbidity present at index date[b], No. (%)**	8,195 (45.2)	956 (38.9)	7,239 (46.1)
Autoimmune disease or immune deficiency	326 (1.8)	31 (1.3)	296 (1.9)
Thyroid disease	286 (1.6)	22 (0.9)	264 (1.7)
COPD	1,696 (9.3)	162 (6.6)	1,534 (9.8)
Asthma and other chronic lung diseases	338 (1.9)	25 (1.0)	313 (2.0)
History of major and clinically relevant bleeding	811 (4.5)	98 (4.0)	713 (4.5)
History of VTE	287 (1.6)	59 (2.4)	228 (1.5)
History of ATE	193 (1.1)	32 (1.3)	161 (1.0)
History of stroke	479 (2.6)	52 (2.1)	427 (2.7)
History of MI	1,666 (9.2)	134 (5.5)	1,532 (9.8)
Anemia	1,440 (7.9)	136 (5.5)	1,304 (8.3)
Coagulopathy	391 (2.2)	38 (1.5)	353 (2.3)
Heart failure	2,380 (13.1)	93 (3.8)	2,287 (14.6)
Valvular heart disease	1,660 (9.1)	108 (4.4)	1,552 (9.9)
Atrial fibrillation	4,332 (23.9)	420 (17.1)	3,912 (24.9)
Atherosclerosis	1,622 (8.9)	161 (6.6)	1,461 (9.3)
Peripheral artery disease	331 (1.8)	41 (1.7)	290 (1.8)
Diabetes mellitus	2,292 (12.6)	198 (8.1)	2,094 (13.3)
Hypertension	3,119 (17.2)	295 (12.0)	2,824 (18.0)
Kidney disease	1,982 (10.9)	138 (5.6)	1,844 (11.8)
Liver disease	247 (1.4)	31 (1.3)	216 (1.4)

Abbreviations: ATE, arterial thrombotic event; COPD, chronic obstructive pulmonary disease; MI, myocardial infarction; VTE, venous thromboembolic event.
Notes: [a]Percentile groups were determined based on disposable income of private households of the complete Dutch population in the Statistics Netherlands database.
[b]Comorbidities were identified by examining data on hospitalizations within 3 years before the index date using ICD-10 codes and ICD-9 codes, restricting to main or primary diagnosis of hospital admission. One or more comorbidities can be present.

 © 2025. The Author(s).

Supplementary Table S9 Cancer-specific baseline characteristics

	Cancer ($N=2{,}457$)
**Differentiation grade[a], No. (%)**	
Grade I	36 (1.5)
Grade II	286 (11.6)
Grade III	380 (15.5)
Grade IV	83 (3.4)
Unknown or not applicable	1672 (68.1)
**Treatment of cancer, No. (%)**	
Chemotherapy	675 (27.5)
Radiotherapy	405 (16.5)
Surgery[b]	321 (13.1)
Hormonal therapy	49 (2.0)
Targeted therapy	119 (4.8)

Notes: [a]The differentiation grade was identified by using the 6th number of the ICD-O-3 codes.
[b]Surgery was defined as any surgical procedure involving the removal of (a portion of) an organ.

Supplementary Table S10 Median survival and follow-up times for different life-limiting diseases

	N at $t=0$	Number of deaths	Median survival time (95%CI)	Median follow-up time (IQR)
**Total**	18,145	10,948	2.03 (1.97, 2.10)	3.59 (1.95, 5.22)
**Type of life-limiting disease**				
Cancer	2,457	2,072	0.35 (0.32, 0.38)	3.41 (2.06, 5.07)
Non-cancer	15,688	8,876	2.50 (2.43, 2.59)	3.60 (1.95, 5.22)
**Type of non-cancer life-limiting disease**				
COPD	547	251	2.10 (1.72, 2.43)	1.93 (1.09, 2.64)
Dementia	387	275	2.14 (1.84, 2.57)	4.75 (2.80, 6.00)
Diabetes mellitus	327	203	2.28 (1.88, 2.94)	4.64 (2.20, 5.69)
Heart disease	10,879	6,185	2.57 (2.46, 2.68)	3.80 (2.07, 5.32)
Hip fracture	3,277	1792	2.53 (2.40, 2.67)	3.20 (1.77, 4.80)
Interstitial lung disease	107	76	2.14 (1.26, 2.71)	3.58 (2.61, 5.62)
Liver disease	164	94	2.09 (1.09, 3.50)	3.57 (1.88, 5.57)

Abbreviations: CI, confidence interval; COPD, chronic obstructive pulmonary disease; IQR, interquartile range.
Note: A life-limiting disease was defined according to the definition of a severe medical condition by Kelley et al as "a diagnosis that carries an increased risk of mortality, hospitalization and emergency room visits."[27] These diseases were identified by ICD-10 codes of diagnoses registered as either main or primary diagnosis of the hospital admission or registered cancer diagnosis by the Netherlands Cancer Registry.
Median survival and follow-up times were estimated by the Kaplan-Meier estimator.

 © 2025. The Author(s).

Supplementary Table S11 Cumulative incidence and incidence rate of VKA and anticoagulant treatment discontinuation stratified by life-limiting disease

	VKA discontinuation				Anticoagulation discontinuation			
	IR/100 PY (95%CI)	6-month cumulative incidence % (95%CI)	1-year cumulative incidence % (95%CI)	3-year cumulative incidence % (95%CI)	IR/100 PY (95%CI)	6-month cumulative incidence % (95%CI)	1-year cumulative incidence % (95%CI)	3-year cumulative incidence % (95%CI)
Cancer	63.1 (59.3– 67.0)	33.7 (31.9–35.6)	38.7 (36.7–40.6)	43.7 (41.7–45.8)	42.6 (39.7– 45.7)	23.8 (22.1–25.5)	28.7 (26.9–30.5)	33.7 (31.7–35.6)
COPD	17.3 (14.1–21.0)	10.8 (8.3–13.7)	13.6 (10.8–16.7)	24.7 (20.3–29.3)	10.4 (8.0–13.2)	6.4 (4.6–8.8)	8.2 (6.0–10.7)	17.3 (13.0–22.1)
Dementia	21.7 (18.4–25.5)	16.8 (13.3–20.7)	23.2 (19.1–27.6)	35.6 (30.7–40.6)	19.9 (16.7–23.4)	15.5 (12.1–19.4)	22.2 (18.2–26.5)	33.5 (28.7–38.3)
Diabetes mellitus	17.1 (13.9–20.7)	12.3 (9.0–16.1)	14.9 (11.2–19.0)	27.0 (22.0–32.1)	14.1 (11.3–17.4)	10.8 (7.7–14.4)	12.7 (9.3–16.6)	23.6 (18.8–28.6)
Heart disease	13.8 (13.3–14.3)	11.0 (10.4–11.6)	14.4 (13.7–15.1)	23.8 (22.9–24.6)	9.4 (9.0–9.8)	7.4 (6.9–7.9)	10.1 (9.5–10.7)	17.4 (16.6–18.2)
Hip fracture	18.7 (17.6–19.9)	14.9 (13.7–16.1)	18.4 (17.1–19.8)	30.1 (28.4–31.9)	15.5 (14.4–16.5)	12.7 (11.6–13.9)	15.8 (14.6–17.1)	26.1 (24.4–27.7)
Interstitial lung disease	18.6 (12.7–26.2)	12.2 (6.8–19.2)	16.9 (10.5–24.7)	29.4 (20.6–38.8)	12.0 (7.5–18.1)	9.4 (4.8–15.9)	11.3 (6.1–18.2)	19.1 (12.0–27.6)
Liver disease	29.6 (22.8–37.9)	22.1 (16.1–28.8)	29.9 (23.0–37.2)	36.5 (28.9–44.1)	21.0 (15.5- 27.7)	17.9 (12.4–24.1)	24.4 (18.0–31.3)	30.5 (23.3–38.0)

Abbreviations: CI, confidence interval; COPD, chronic obstructive pulmonary disease; IR, incidence rate; PY, person-years; VKA, vitamin K antagonist.
Note: Cumulative incidences were computed taking the competing risk of death into account. Crude incidence rates (IR) were estimated as events per 100 person-years (PY).

Supplementary Table S12 Mean proportion of days covered with anticoagulants during follow-up

	Number of patients, No.	Mean PDC % (SD)	Adherent patients[a] %
**Total**	18,145	91.8 (22.2)	88.0
**Type of life-limiting disease**			
Cancer	2,457	87.2 (26.4)	81.0
Non-cancer	15,688	92.6 (21.4)	89.2
**Type of non-cancer life-limiting disease**			
COPD	547	95.3 (17.3)	93.6
Dementia	387	83.9 (30.2)	75.5
Diabetes mellitus	327	88.0 (26.4)	82.9
Heart disease	10,879	94.1 (18.8)	91.3
Hip fracture	3,277	88.9 (26.3)	84.3
Interstitial lung disease	107	92.7 (21.2)	87.9
Liver disease	164	82.3 (32.2)	76.8
**Sex**			
Females	8,841	91.0 (23.4)	86.8
Males	9,304	92.6 (21.1)	89.2

Abbreviations: COPD, chronic obstructive pulmonary disease; PDC, proportion of days covered; SD, standard deviation.
Note: [a]Adherent was defined as a proportion of days covered (PDC) with anticoagulants >80%.

 © 2025. The Author(s).

Supplementary Table S13 Cumulative incidence and incidence rate of VKA and anticoagulant treatment discontinuation in sensitivity analysis of VKA exposure time

	VKA discontinuation				Anticoagulation discontinuation			
	IR / 100 PY (95%CI)	6-month cumulative incidence % (95%CI)	1-year cumulative incidence % (95%CI)	3-year cumulative incidence % (95%CI)	IR / 100 PY (95%CI)	6-month cumulative incidence % (95%CI)	1-year cumulative incidence % (95%CI)	3-year cumulative incidence % (95%CI)
**Total**	17.7 (17.2–18.2)	14.9 (14.4–15.5)	18.6 (18.1–19.2)	27.7 (27.0–28.4)	12.7 (12.3–13.1)	10.8 (10.4–11.3)	14.0 (13.5–14.5)	21.4 (20.7–22.0)
Type of life-limiting disease								
Cancer	62.8 (59.0–66.7)	33.7 (31.8–35.5)	38.5 (36.6–40.5)	43.6 (41.6–45.6)	42.4 (39.5–45.4)	23.7 (22.0–25.4)	28.5 (26.7–30.4)	33.5 (31.6–35.5)
Non-cancer	15.0 (14.5–15.5)	12.0 (11.5–12.5)	15.5 (15.0–16.1)	25.3 (24.5–26.0)	10.8 (10.4–11.2)	8.8 (8.4–9.3)	11.7 (11.2–12.2)	19.5 (18.8–20.2)
Type of non-cancer life-limiting disease								
COPD	17.1 (14.0–20.8)	10.6 (8.2–13.4)	13.4 (10.6–16.5)	24.5 (20.1–29.2)	10.2 (7.9–13.0)	6.3 (4.4–8.5)	8.0 (5.8–10.5)	17.2 (12.9–22.0)
Dementia	21.7 (18.4–25.5)	16.8 (13.3–20.7)	23.2 (19.1–27.6)	35.6 (30.7–40.6)	19.9 (16.7–23.4)	15.5 (12.1–19.4)	22.2 (18.2–26.5)	33.5 (28.7–38.3)
Diabetes mellitus	16.7 (13.6–20.3)	12.3 (9.0–16.1)	14.9 (11.2–19.0)	26.6 (21.7–31.8)	13.7 (11.0–17.0)	10.8 (7.7–14.4)	12.7 (9.3–16.6)	23.2 (18.5–28.2)
Heart disease	13.5 (13.0–14.1)	10.9 (10.3–11.5)	14.3 (13.7–15.0)	23.4 (22.5–24.2)	9.2 (8.8–9.6)	7.4 (6.9–7.9)	10.1 (9.5–10.7)	17.0 (16.3–17.8)
Hip fracture	18.5 (17.4–19.7)	14.8 (13.6–16.1)	18.3 (16.9–19.6)	29.8 (28.1–31.5)	15.2 (14.2–16.3)	12.6 (11.5–13.8)	15.7 (14.5–17.0)	25.7 (24.1–27.4)
Interstitial lung disease	18.0 (12.2–25.6)	11.3 (6.1–18.1)	16.9(10.5–24.7)	28.1 (19.5–37.4)	11.4 (7.1–17.5)	8.4 (4.1–14.7)	11.3 (6.1–18.2)	17.8 (11.0–26.0)
Liver disease	29.6 (22.7–37.8)	22.1 (16.1–28.8)	29.9 (23.0–37.2)	36.5 (28.9–44.2)	21.0 (15.5–27.7)	17.9 (12.4–24.1)	24.4 (18.0–31.3)	30.5 (23.3–38.0)
Sex								
Females	17.8 (17.1–18.5)	14.9 (14.1–15.6)	18.9 (18.0–19.7)	28.7 (27.7–29.7)	13.1 (12.6–13.7)	11.1 (10.4–11.7)	14.4 (13.6–15.1)	22.6 (21.7–23.6)
Males	17.6 (16.9–18.3)	15.0 (14.3–15.7)	18.4 (17.6–19.2)	26.8 (25.9–27.8)	12.3 (11.7–12.9)	10.6 (10.0–11.3)	13.6 (12.9–14.3)	20.2 (19.4–21.1)

Abbreviations: CI, confidence interval; COPD, chronic obstructive pulmonary disease; IR, incidence rate; PY, person-years; VKA, vitamin K antagonist.
Note: This table displays the cumulative incidences and incidence rates of vitamin K antagonist (VKA) and anticoagulant treatment discontinuation calculated in a sensitivity analysis where we varied the exposure time of a VKA prescription when constructing treatment periods (150 days + 100 days added versus 180 days + 60 days added in the main analysis).
Cumulative incidences were computed taking the competing risk of death into account. Crude incidence rates (IR) were estimated as events per 100 person-years (PY).

 © 2025. The Author(s).

Supplementary Table S14 Cumulative incidence and incidence rate of anticoagulant treatment discontinuation in sensitivity analysis of heparin exposure time

Anticoagulation discontinuation				
	IR/100 PY (95%CI)	6-month cumulative incidence % (95%CI)	1-year cumulative incidence % (95%CI)	3-year cumulative incidence % (95%CI)
**Total**	12.7 (12.3–13.1)	10.7 (10.3–11.2)	13.8 (13.3–14.3)	21.2 (20.6–21.9)
**Type of life-limiting disease**				
Cancer	39.9 (37.1–42.8)	22.9 (21.3–24.6)	27.2 (25.5–29.0)	31.4 (29.5–33.3)
Non-cancer	10.9 (10.6–11.3)	8.8 (8.4–9.3)	11.7 (11.2–12.2)	19.7 (19.0–20.3)
**Type of non-cancer life-limiting disease**				
COPD	10.1 (7.8–12.9)	6.4 (4.6–8.8)	7.9 (5.8–10.5)	15.9 (12.2–20.1)
Dementia	19.9 (16.7–23.4)	15.5 (12.1–19.4)	22.2 (18.2–26.5)	33.5 (28.7–38.3)
Diabetes mellitus	13.9 (11.1–17.2)	10.8 (7.7–14.4)	12.7 (9.3–16.6)	22.8 (18.1–27.7)
Heart disease	9.3 (8.9–9.7)	7.3 (6.9–7.8)	10.0 (9.5–10.6)	17.2 (16.5–18.0)
Hip fracture	15.4 (14.4–16.4)	12.7 (11.6–13.9)	15.7 (14.5–17.0)	26.0 (24.3–27.6)
Interstitial lung disease	12.0 (7.5–18.1)	9.4 (4.8–15.9)	11.3 (6.1–18.2)	19.1 (12.0–27.6)
Liver disease	20.9 (15.5–27.7)	17.9 (12.4–24.1)	24.4 (18.0–31.3)	29.5 (22.5–36.9)
**Sex**				
Females	13.1 (12.6–13.7)	11.0 (10.4–11.7)	14.3 (13.6–15.1)	22.5 (21.6–23.5)
Males	12.2 (11.6–12.8)	10.5 (9.9–11.1)	13.3 (12.6–14.0)	20.0 (19.2–20.9)

Abbreviations: CI, confidence interval; COPD, chronic obstructive pulmonary disease; IR, incidence rate; PY, person-years.
Note: This table displays the cumulative incidences and incidence rates of anticoagulant treatment discontinuation calculated in a sensitivity analysis where we varied the exposure time of a heparin (i.e., LMWH) prescription when constructing treatment periods (100 days added versus 30 days in the main analysis).
Cumulative incidences were computed taking the competing risk of death into account. Crude incidence rates (IR) were estimated as events per 100 person-years (PY).

Supplementary Table S15 Incidence rates of bleeding and thromboembolic events stratified by anticoagulation exposure in sensitivity analyses

	Extending AC exposed period by 7 days		Sensitivity analysis of heparin exposure time		Sensitivity analysis of VKA exposure time	
	IR/100 PY AC exposed (95%CI)	IR/100 PY unexposed (95%CI)	IR/100 PY AC exposed (95%CI)	IR/100 PY unexposed (95%CI)	IR/100 PY AC exposed (95%CI)	IR/100 PY unexposed (95%CI)
**Major and clinically relevant bleeding**	2.6 (2.4–2.8)	1.9 (1.4–2.7)	2.6 (2.4–2.8)	2.1 (1.5–2.8)	2.6 (2.4–2.8)	2.0 (1.5–2.7)
**Venous thromboembolism**	0.2 (0.1–0.2)	0.4 (0.2–0.8)	0.2 (0.1–0.2)	0.4 (0.2–0.7)	0.2 (0.1–0.2)	0.4 (0.2–0.8)
**Arterial thromboembolism**	3.1 (2.9–3.3)	3.0 (2.3–3.8)	3.1 (2.9–3.3)	3.2 (2.5–4.1)	3.1 (2.9–3.3)	3.3 (2.5–4.1)
**Myocardial infarction**	1.1 (1.0–1.3)	0.8 (0.5–1.3)	1.1 (1.0–1.3)	0.8 (0.5–1.3)	1.1 (1.0–1.3)	0.9 (0.5–1.4)
**Stroke**	1.6 (1.5–1.7)	1.8 (1.3–2.4)	1.6 (1.4–1.7)	2.0 (1.5–2.7)	1.6 (1.4–1.7)	2.0 (1.4–2.7)
**Other**	0.4 (0.4–0.5)	0.5 (0.2–0.8)	0.4 (0.4–0.5)	0.4 (0.2–0.8)	0.4 (0.4–0.5)	0.5 (0.2–0.9)

Abbreviations: AC, anticoagulant; CI, confidence interval; IR, incidence rate; PY, person-years.
Note: This table displays the incidence rates of first bleeding and thromboembolic events stratified by anticoagulant exposure, estimated in three sensitivity analyses. The following sensitivity analyses were performed: (1) extending the period exposed to anticoagulants by 7 days, (2) varying the exposure time of a heparin (i.e., LMWH) prescription when constructing treatment periods (100 days added versus 30 days in the main analysis), (3) varying the exposure time of a VKA prescription when constructing treatment periods (150 days + 100 days added versus 180 days + 60 days added in the main analysis). Incidence rates were estimated as events per 100 PYs, where the observation time was categorized according to anticoagulant exposure.

 © 2025. The Author(s).

References

1 Persoonskenmerken van alle in de Gemeentelijke Basis Administratie (GBA) ingeschreven personen, gecoördineerd. Version V1. ODISSEI Portal; 2021. Accessed at: https://doi.org/10.57934/0b01e4108071ba40

2 Chen Q, van Rein N, van der Hulle T, et al. Coexisting atrial fibrillation and cancer: time trends and associations with mortality in a nationwide Dutch study. Eur Heart J 2024;45(25):2201–2213

3 Inkomen van huishoudens (revisie 2017). Version V1. ODISSEI Portal; 2011. Accessed at: https://doi.org/10.57934/0b01e41080371196

4 Datum van overlijden van personen die ingeschreven staan in de Gemeentelijke Basisadministratie (GBA). Version V1. ODISSEI Portal; 2018. Accessed at: https://doi.org/10.57934/0b01e410803b37dc

5 Doodsoorzaken van personen die bij overlijden inwoners waren van Nederland. Version V1. ODISSEI Portal; 2013. Accessed at: https://doi.org/10.57934/0b01e410802359a7

6 Diagnosen behorend bij ziekenhuisopnamen Landelijke Basisregistratie Ziekenhuiszorg. Version V1. ODISSEI Portal; 2019. Accessed at: https://doi.org/10.57934/0b01e410805d9385

7 Ziekenhuisopnamen Landelijke Basisregistratie Ziekenhuiszorg. Version V1. ODISSEI Portal; 2019. Accessed at: https://doi.org/10.57934/0b01e410805d96a7

8 Ziekenhuisopnamen voor RA-gebruik. Version V1. ODISSEI Portal; 2012. Accessed at: https://doi.org/10.57934/0b01e4108030bccb

9 Diagnosen behorend bij ziekenhuisopnamen voor RA-gebruik. Version V1. ODISSEI Portal; 2012. Accessed at: https://doi.org/10.57934/0b01e4108030be8c

10 Chen Q, Toorop MMA, Tops LF, Lijfering WM, Cannegieter SC. Time trends in patient characteristics, anticoagulation treatment, and prognosis of incident nonvalvular atrial fibrillation in the Netherlands. JAMA Netw Open 2023;6(04):e239973

11 Verstrekkingen van geneesmiddelen op 4 posities ATC-code aan personen. Version V1. ODISSEI Portal; 2020. Accessed at: https://doi.org/10.57934/0b01e41080757f4a

12 Wickham H, Averick M, Bryan J, et al. Welcome to the tidyverse. J Open Source Softw 2019;4(43):1686

13 Wickham H, François R, Henry L, Müller K, Vaughan D. dplyr: A Grammar of Data Manipulation. R package version 1.1.4. Accessed May 27, 2024 at: https://CRAN.R-project.org/package=dplyr

14 Grolemund G, Wickham H. Dates and times made easy with lubridate. J Stat Softw 2011;40(03):1–25

15 Wickham H. stringr: simple, consistent wrappers for common string operations. R package version 1.5.1. Accessed August 13, 2024 at: https://CRAN.R-project.org/package=stringr

16 Wickham H, Vaughan D, Girlich M. tidyr: tidy messy data. R package version 1.3.1. Accessed August 13, 2024 at: https://CRAN.R-project.org/package=tidyr

17 R Core Team. foreign: read data stored by "Minitab," "S," "SAS," "SPSS," "Stata," "Systat," "Weka," "dBase," ... R package version 0.8–86. Accessed August 13, 2024 at: https://CRAN.R-project.org/package=foreign

18 Wickham H. forcats: tools for working with categorical variables (factors). R package version 1.0.0. Accessed August 13, 2024 at: https://CRAN.R-project.org/package=forcats

19 Wickham H, Henry L. purrr: functional programming tools. R package version 1.0.2. Accessed August 13, 2024 at: https://CRAN.R-project.org/package=purrr

20 Wickham H, Chang W, Henry L, et al. ggplot2: create elegant data visualisations using the grammar of graphics. Accessed September 20, 2022 at: https://CRAN.R-project.org/package=ggplot2

21 Therneau T. A package for survival analysis in R. R package version 3.6–4. Accessed August 13, 2024 at: https://CRAN.R-project.org/package=survival

22 Gerds TA. prodlim: product-limit estimation for censored event history Analysis. R package version 2023.08.28. Accessed August 13, 2024 at: https://CRAN.R-project.org/package=prodlim

23 Stevenson M, Sergeant E. epiR: tools for the analysis of epidemiological data. R package version 2.0.74. Accessed August 13, 2024 at: https://cran.r-project.org/package=epiR

24 Sjoberg DD, Fei T. tidycmprsk: competing risks estimation. R package version 1.0.0. Accessed August 13, 2024 at: https://CRAN.R-project.org/package=tidycmprsk

25 Sjoberg D, Baillie M, Fruechtenicht C, Haesendonckx S, Treis T. ggsurvfit: flexible time-to-event figures. R package version 1.1.0. Accessed August 13, 2024 at: https://CRAN.R-project.org/package=ggsurvfit

26 Wilke C. cowplot: streamlined plot theme and plot annotations for "ggplot2." R package version 1.1.3. Accessed August 13, 2024 at: https://CRAN.R-project.org/package=cowplot

27 Kelley AS, Ferreira KB, Bollens-Lund E, Mather H, Hanson LC, Ritchie CS. Identifying older adults with serious illness: transitioning from ICD-9 to ICD-10. J Pain Symptom Manage 2019;57(06):1137–1142

 © 2025. The Author(s).
